# Trivalent rare earth metal cofactors confer rapid NP-DNA polymerase activity

**DOI:** 10.1126/science.adh5339

**Published:** 2023-10-26

**Authors:** Victor S. Lelyveld, Ziyuan Fang, Jack W. Szostak

**Affiliations:** 1 Howard Hughes Medical Institute, Massachusetts General Hospital, Boston, MA, 02114 USA.; 2 Center for Computational and Integrative Biology, Department of Molecular Biology, Massachusetts General Hospital, Boston, MA, 02114 USA.; 3 Department of Genetics, Harvard Medical School, Boston, MA, USA.; 4 Department of Chemistry and Chemical Biology, Harvard University, Cambridge, MA, USA.; 5 Howard Hughes Medical Institute, The University of Chicago, Chicago, IL, 60637 USA.; 6 Department of Chemistry, University of Chicago, Chicago, IL, 60637 USA.

## Abstract

A DNA polymerase with a single mutation and divalent calcium cofactor catalyzes the synthesis of unnatural N3′→P5′ phosphoramidate (NP) bonds to form NP-DNA. However, this template-directed phosphoryl transfer activity remains orders-of-magnitude slower than native phosphodiester synthesis. Here we used time-resolved X-ray crystallography to show that NP-DNA synthesis proceeds with a single detectable calcium ion in the active site. Using insights from isotopic and elemental effects, we propose that one-metal-ion electrophilic substrate activation is inferior to the native two-metal-ion mechanism. We find that this deficiency in divalent activation could be ameliorated by trivalent rare earth and post-transition metal cations, dramatically enhancing NP-DNA synthesis. Scandium(III), in particular, confers highly specific NP activity with kinetics enhanced by >100-fold over calcium(II), yielding NP-DNA strands ≥100 nts in length.

## One-Sentence Summary

A DNA polymerase catalyzes rapid unnatural bond synthesis when furnished with trivalent rare earth metal cofactors.

The principal chemistry at the core of RNA and DNA metabolism is phosphodiester synthesis. Polymerases generate nascent strands of genetic material by stepwise phosphoryl transfer of nucleotides to primer termini, yielding O3′→P5′ phosphodiester linkages. This template-directed process is conserved across all known biology. Nucleotide 3′ substitutions have therefore been widely regarded as chain terminating for polymerase activity, forming the basis for a class of nucleoside analog drugs (1, 2).

**Fig. 1.**
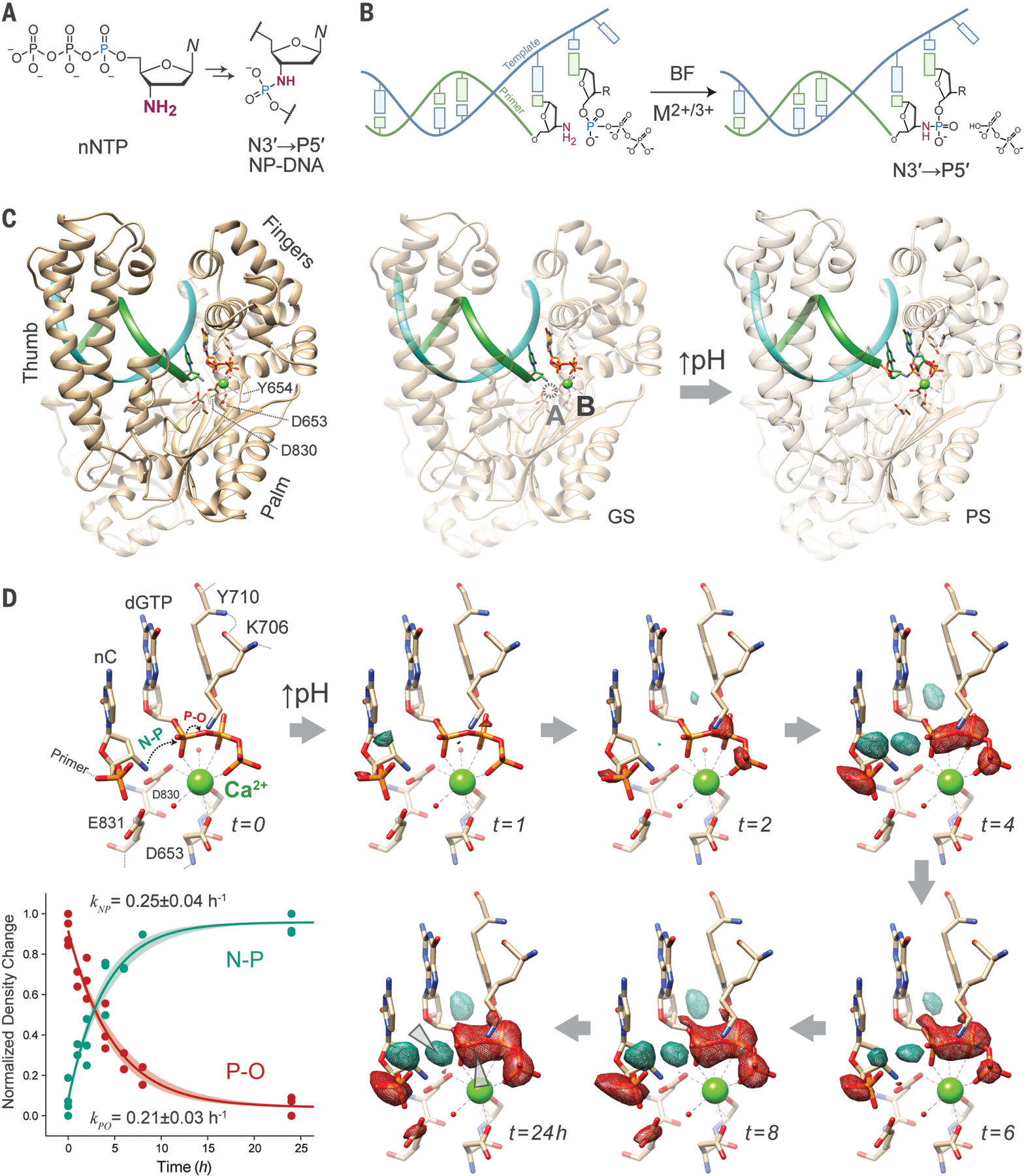
N3′→P5′ (NP) polymerase activity proceeds in crystallo by a one-metal-ion mechanism. (**A**) Condensation of 3′-amino 2′,3′-dideoxynucleoside 5′-triphosphates (nNTPs) generates N3′→P5′ phosphoramidate DNA (NP-DNA). (**B**) Reaction scheme for BF-catalyzed primer extension of a 3′-amino terminal primer on a DNA template, yielding pyrophosphate and an NP-linked +1 nucleotide extension. R = OH for in-crystallo single nucleotide addition of dGTP with co-crystallized divalent metal ion cofactor Ca^2+^, whereas R = NH_2_ for multiple turnover extension in solution to synthesize NP-DNA polynucleotide strands in the presence of divalent (M^2+^) or trivalent (M^3+^) metal ion cofactors. (**C**) Ribbon cartoons of BF polymerase ground state (GS) holocomplex X-ray crystal structure with 3′-amino terminal DNA primer (green ribbon) and DNA template (cyan) with bound dGTP substrate and a single Ca^2+^ ion (green atom) in the canonical metal-B site (left and middle panels), showing one of two complexes co-crystallized in the asymmetric unit. All analyses performed here derive from the first complex (chains A-C), but consistent trends are seen in both. Dotted circle indicates the position of a missing A-site metal in analogous models of the canonical phosphodiester synthesis mechanism but not observed in closed conformation GS structures containing a 3′-NH_2_ (middle panel) or product state (PS) structures of the in-situ synthesized N3′→P5′ phosphoramidate (NP) bond. (**D**) Representative time-dependent difference maps (*F*_*o*_ *– F*_*c*_) showing density changes in the BF F710Y/D598A active site during NP bond formation in crystallo. GS crystals containing a 3′-amino-2′,3′-dideoxycytidine (nC) terminated primer and DNA template were formed under acidic conditions (pH 5 – 6) in the presence of Ca^2+^ and dGTP, and the in situ reaction was initiated by transferring crystals to a solution at pH 8.8. The reaction in intact crystals was quenched at the indicated times by flash freezing for subsequent data collection. Positive (aquamarine mesh) or negative (red mesh) isosurfaces are shown contoured at 2.5 σ, superimposed on the GS model. Bottom-left inset: normalized density change extracted from difference maps at the nascent N-P (aquamarine) and scissile O-P (red) bonds quantified from 19 crystals (dots), observed first-order rate constant estimates for the density changes ± s.d. plotted (solid line ± shaded area).

In recent work, we demonstrated the direct enzymatic synthesis of an unnatural linkage by substitution of the 3′-OH nucleophile with a 3′-amine, extending the chemistry amenable to polymerase catalysis (1). We reported that 3′-NH_2_ primer extension can be catalyzed by a modified DNA polymerase, yielding N3′→P5′ phosphoramidate DNA (NP-DNA, Fig. 1A,B). The large fragment of DNA polymerase I (BF), cloned from the thermophilic soil bacterium *Geobacillus stearothermophilus* (Bst), acquires nontrivial levels of N3′→P5′ polymerase activity via two surprisingly minor substitutions: (A) a single active site mutation (F710Y) and (B) substitution of its divalent Mg^2+^ cofactors with Ca^2+^ (1). However, this level of activity remained around four orders-of-magnitude slower than native phosphodiester synthesis. Although phosphoramidate (NP) synthesis was detectable in the presence of several divalent alkaline earth metal ions, the pattern of metal cofactor activity was distinct from that found in native phosphodiester activity (3). Crystal structures of the ground-state NP reaction complex showed a single metal ion in the active site (1), suggesting a plausible distinction between the NP mechanism and the classical two-metal-ion mechanism in native phosphodiester activity (4). Whether NP catalysis does in fact rely on a distinct mechanism with a nonclassical cofactor configuration remains to be established, as do the critical factors limiting the kinetics of NP synthesis.

## Linearly correlated density dynamics in reacting BF crystals during NP synthesis

To understand the mechanistic barriers to rapid enzymatic NP-DNA synthesis, we first sought to observe NP bond formation through crystallography. In previous x-ray crystal structures of the fully assembled NP polymerase pre-insertion complex, we modeled a single divalent metal ion in the reaction center, occupying a site distal to the primer-terminal nucleophile (1). This site is equivalent to the so-called “B site” in the classical two-metal-ion reaction center and includes inner sphere ligands from the substrate triphosphate moiety, the side chains of aspartates 830 and 653, and the backbone amide of Tyr-654. No evidence for an “A-site” metal proximal to the primer 3′-amino terminus was observed in these earlier pre-chemistry model structures, a finding consistent with the expectation that a neutral amine is the nucleophile in NP synthesis. However, earlier structures did not fully recapitulate the active NP reaction center or the product state (1). The A-site metal ion can also be poorly ordered even in structures of the native polymerase reaction complex. Nakamura *et al*. reported that accumulation of an additional metal ion in a “C site” of polymerase η, a polymerase Y family member, occurs in a manner linearly correlated with bond formation, suggesting that the product state’s post-chemistry metal configuration may be distinct from that observed in the pre-chemistry reaction complex (5). This observation was subsequently extended to polymerase X family members (6, 7). To add evidence that a single divalent metal ion cofactor catalyzes unnatural NP synthesis in BF, a polymerase A family member, we carried out a single nucleotide primer extension reaction in intact crystals (Fig. 1B–D). We crystallized active substrate-bound BF polymerase F710Y/D598A with a 3′-amino terminal DNA primer and DNA template in the presence of Ca^2+^ and 2′-deoxyguanosine 5′-triphosphate (dGTP). Reactions were then initiated in the intact crystals by pH-shift from an acidic mother liquor (pH < 6) to a basic soaking liquor at pH 8.8, and reacting crystals were quenched at various times by flash freezing (Fig. 1C,D).

Quantitative analysis of electron density dynamics is complicated by crystal-to-crystal variance in diffraction datasets. We therefore incorporated datasets arising from up to 4 crystals at each of 6 time points following initiation. By scaling reflections from 19 total crystals (resolution 2.0 – 2.7 Å) across the time course (0 – 24 h), we could estimate voxel-wise first-order rate constants for changes in real-space electron density across the asymmetric unit and particularly at the reaction center (Fig. 1D, bottom left panel). Early time points show negligible differences in ordered density between the ground state complex crystallized under acidic conditions versus the complex after 1 h of soaking under reaction conditions, suggesting that the reactant state and ground state cannot be clearly distinguished in this resolution range. By following the reaction at subsequent times, we observed monotonic accumulation of nascent NP bond density between the primer terminal 3′-amino group and the substrate α-phosphate with concomitant loss of electron density between the α- and β-phosphates with similar first-order rate constants (Fig. 1D).

**Fig. 2.**
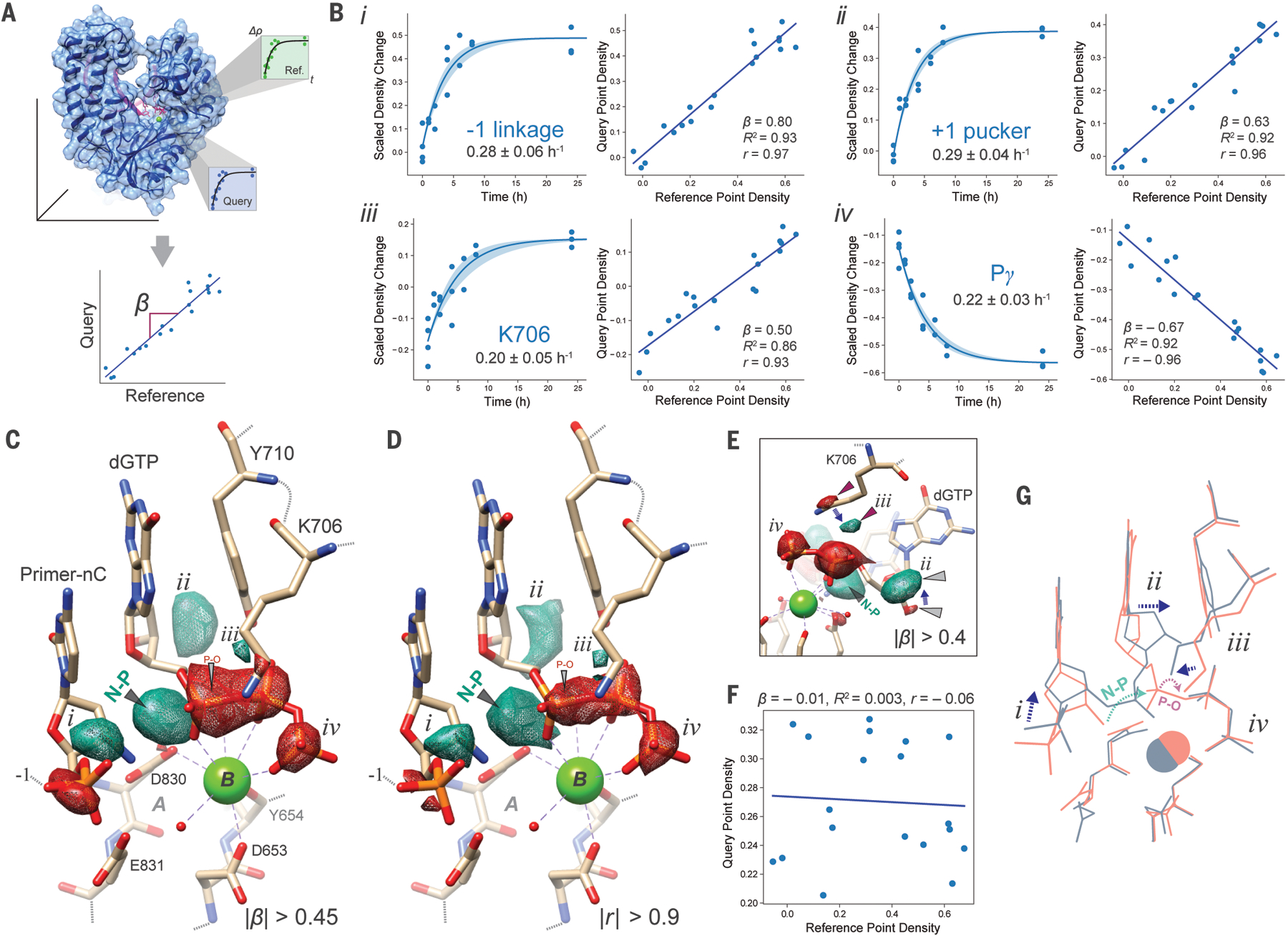
One-metal-ion NP catalysis is limited by deficient substrate activation. (**A**) Voxel-wise linear regression analysis of density dynamics in crystallo. Difference map (*F*_*o*_ *– F*_*c*_) densities derived from reacting crystals quenched at various times yield kinetic traces at a reference point (Ref.) or any query point of interest. A pairwise linear regression slope, *β*, of density changes at the reference vs. query points can be estimated for any two points in real space. (**B**) Regression statistics of key density dynamics during N3′→P5′ synthesis in BF F710Y/D598A. Insets *i – iv* show difference map density derived from individual crystals at four major active-site blobs indicated in panel C. Left panels: first-order kinetics of density differences from individual crystals (dots) over reaction time, with values for the estimated rate constant ± s.d. plotted (solid line ± shaded area). Right panels: pairwise linear regressions, coefficients of determination (*R*^*2*^), and correlation coefficients (*r*) of difference map densities at the indicated site vs. the NP bond across all crystals. (**C**) Pairwise linear regression “beta” map calculated at all points vs. the nascent NP bond (black wedge) with positive (aquamarine mesh) or negative (red mesh) regression slopes, *β*, displayed as isosurfaces superimposed on the ground state structure, contoured at |*β*| > 0.45. (**D**) Pearson correlation map of the active site contoured at |*r*| > 0.9 calculated by pairwise comparison of all voxels vs. the peak of the nascent bond (black wedge), with positive (aquamarine mesh) or negative (red mesh) correlation displayed as isosurfaces superimposed on the ground state structure. (**E**) Alternative view of the beta map contoured at |*β*| > 0.4, highlighting positive and negative beta peaks at sites (purple wedges) associated with a conformational change at the side chain of Lys-706 (positive lobe quantified in panel B, inset *iii*) in the direction of the substrate O_α,β_ bridging position and the scissile bond, as well as the conformation change in the dGTP substrate sugar (grey wedges, positive lobe quantified in panel B, inset *ii*). (**F**) Pairwise linear regression and correlation statistics vs. the nascent bond calculated at a position equivalent to the absent A-site metal (grey ‘A’ label in panels C,D). (**G**) Cartoon overlay of ground state (peach) and product state (blue) models with indicated conformational dynamics labeled as in panels B-D.

By inspecting the kinetics of density changes at nearby sites, we noticed that the most prominent local density dynamics in the active site had simple linear relationships with nascent bond formation. Pairwise linear regression yields the relative slope, *β*, of density changes occurring at any two points across the time-resolved dataset (Fig. 2A,B). Performing this regression at all points in the asymmetric unit vs. the nascent bond furnished a field of *β* values or “beta map” in real space, which could be contoured at a desired threshold of |*β*| to give isosurfaces for visualization (Fig. 2C). To show that regions with high |*β*| are also highly correlated, we produced pairwise Pearson correlation maps by a similar procedure (Fig. 2D). Regions of local conformational dynamics were qualitatively in agreement between correlation maps with coefficient, *>r*, contoured at |*r*| > 0.9 (Fig. 2D) vs. regression maps contoured at |*β*| > 0.45 (Fig. 2C). When beta maps were contoured at |*β*| > 0.9, we observed that the included voxels were tightly constrained to the site of the nascent NP bond and the scissile P_α_–O_α,β_ bond (Fig. S1A). No other linear density dynamics are larger in scalar magnitude than the chemistry itself during bond formation in crystallo.

Inspecting the isosurfaces contoured at |*β*| > 0.45, we observed four major density perturbations that are also highly correlated (|*r*| > 0.9) with the nascent bond (Fig. 2B, insets *i - iv*): (*i*) displacement of the terminal phosphodiester linkage between the −1 position and the primer terminus, (*ii*) a conformational switch of the substrate deoxyribose moiety from C2′-endo to C3′-endo, matching the sugar pucker of the primer terminus, (*iii*) a small deflection of the K706 side chain toward the substrate bridging α,β-oxygen, homologous to a putative general acid in the native mechanism (8), and finally (*iv*) disordering of the leaving group pyrophosphate moiety. As apparent from the high correlation coefficients, maps generated as 1−*p* for two-tailed p-values determined for the correlation field vs. the nascent bond show that all of these active site dynamics are highly significant with *p* < 10^−6^ (Fig. S1B). Deflection of K706 toward the substrate α,β-bridging oxygen is well-resolved on the beta map as positive and negative lobes surrounding terminal atoms of the side chain (Fig. 2E). This motion is noteworthy because the site is structurally homologous to the C-site metal-ion density reported for X- and Y-family polymerases (5–7, 9). Crucially, no well-correlated density changes were observed at a position equivalent to the absent A-site metal in the native mechanism (Fig. 2F).

Conformational changes in both the upstream and downstream nucleotides flanking the nascent bond are linearly correlated with covalent bonding (Fig. 2B insets *i* and *ii*, and panels C,D). The upstream conformational change at the primer-terminal linkage is also observable in time-resolved datasets of native phosphodiester synthesis in polymerase η (5). The downstream conformational change in the dGTP substrate sugar from C2′- to C3′-endo upon product formation (Fig. 2B inset *ii* and Fig. 2E), however, was not observed in an equivalent time-resolved experiment with the wild-type enzyme, in which the conformation is stably C3′-endo (Fig. S2). Although intriguing, a ground state conformational change in the deoxynucleotide substrate is unlikely to be relevant to 3′-amino nucleotides, which already prefer the C3′-endo conformation due to a mixture of steric and anomeric effects (see SI text) (10).

## Isotopic and elemental substitution effects point toward rate-limiting chemistry with Ca^2+^

**Fig. 3.**
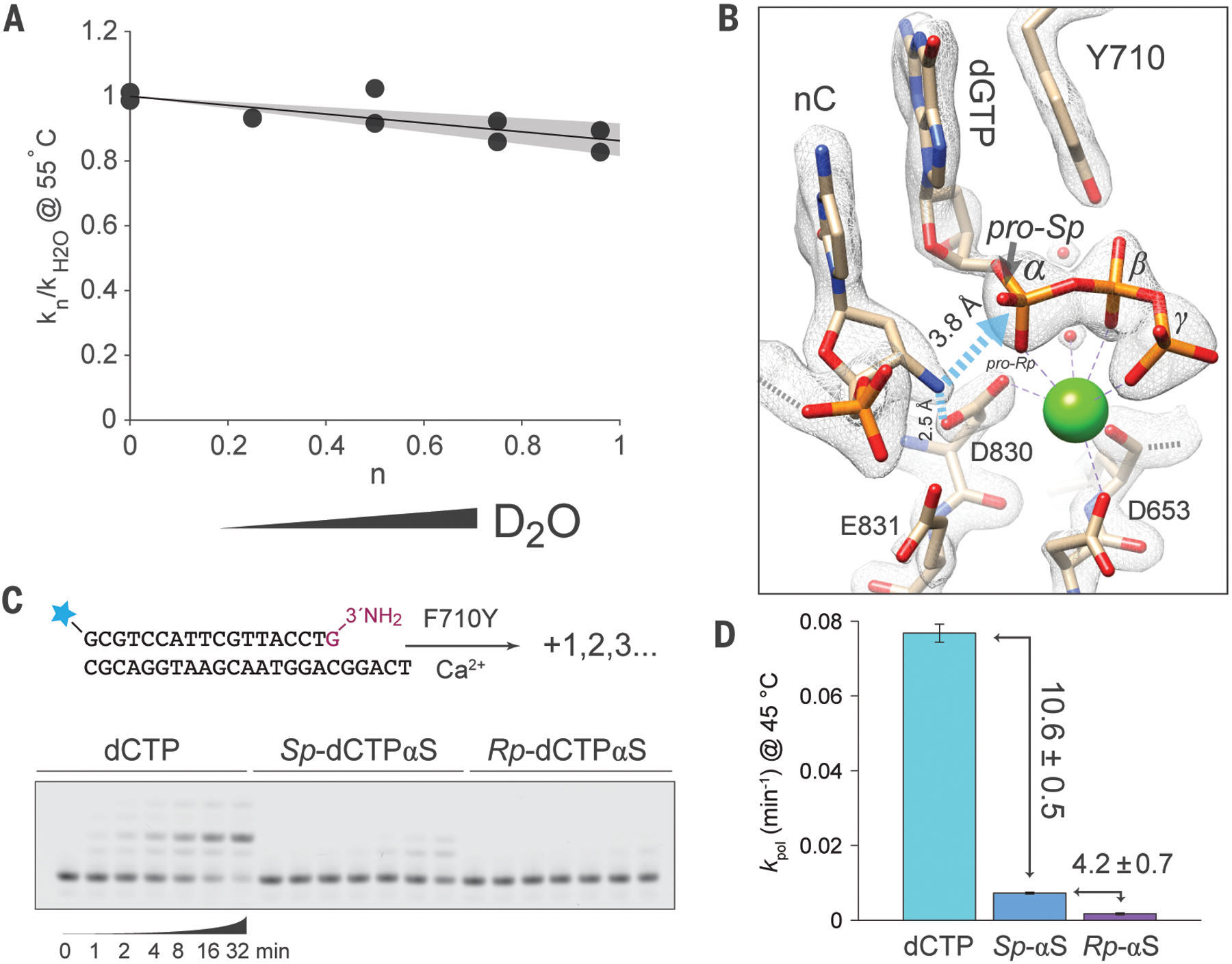
Probing for rate-limiting chemistry in NP-DNA synthesis in solution. (**A**) Pre-steady-state solvent deuterium kinetic isotope effect estimated from the slope of the line k_n_/k_H2O_ = 1 + n(φ−1) for 3′-amino primer extension with 1 mM dCTP and 10 mM CaCl_2_ in varying mole fractions, *n*, of D_2_O. The quantity 1/φ is the SDKIE, estimated at 1.16 with 95% confidence interval (1.09, 1.23) in the shaded region. (**B**) Atomic model distances in the ground state reaction complex for the primer-terminal (nC) 3′-amine to the D830 side chain or to the substrate P_α_. Grey mesh overlay indicates *2F*_*o*_ *- F*_*c*_ density map contoured at 2.5 σ. (**C**) Effect of α-phosphorothioate substitution on pre-steady-state Ca^2+^-activated NP synthesis at 45 °C with 500 μM *Sp*-αS, *Rp*-αS, or unmodified dCTP substrates and BF F710Y. Inset: fluorescently-labeled primer and template strand for the experiments in panels A, C, and D. Representative 15% TBE-urea PAGE separation of quenched reaction samples is shown. Only the first addition to this primer forms an NP or NPS linkage in the presence of 2′-deoxyribose substrates, yielding subsequent phosphodiester or phosphorothioate products, including mismatch extension products. (**D**) Elemental thio effects (labeled arrows, mean ± s.d.) estimated from the pre-steady-state rate constants (*k*_*pol*_, error bars indicate s.d., n = 3) for reactions with substrates as in panel C.

Reacting crystals are sampled on a timescale vastly slower than that of conformational dynamics like furanose pseudorotation. The crystal data is therefore not directly informative of the relative sequence and magnitude of these reaction barriers. To gain more insight into the rate-limiting step for NP synthesis, we turned to solution-phase kinetics. Two proton transfers have been detected in the rate-limiting step of native polymerase activity using careful measurements of the solvent deuterium kinetic isotope effect (SDKIE) (8, 11). In NP synthesis, it is minimally required that one proton arising from the 3′-amino nucleophile must ultimately be transferred out of the reaction center in the forward reaction, yielding the product phosphoramidate. The conserved Asp-830 side chain, proximal to the nucleophilic primer 3′-amine at ~2.5 Å in refined ground state structures (Fig. 3B), is the most likely general base mediating this proton transfer. A sterically-conservative point mutation at this position, D830N, entirely abolishes activity (1). We found that the effect of varying mole fraction, *n*, of D_2_O solvent on pre-steady-state NP reaction kinetics was negligible, yielding an SDKIE estimate of 1.16 for 3′-amino primer extension in the presence of Ca^2+^ and dCTP (Fig. 3A). In native phosphodiester polymerases, this value has generally been measured in the 2 – 5 range (8). The substantially lower value measured here suggests that proton transfer is not a critical barrier in the NP reaction with Ca^2+^.

If the chemical step of NP bond synthesis sets the overall reaction rate, then modulating the electrophilicity of the substrate should show an appreciable kinetic effect. We therefore measured the non-bridging elemental thio-substitution effect in solution using stereopure *Sp* or *Rp* diastereomers of dCTPαS. We found that the thio effect was k_O_/k_S_ = 10.6 ± 0.5 (mean ± s.d.) when substituting the dCTP substrate with *Sp*-dCTPαS in pre-steady-state extension of a 3′-amino terminus at 45 °C (Fig. 3C,D). Elemental thio effects of similar magnitude have been observed for native polymerase mismatch extension or when the divalent cofactors are Mn^2+^ rather than Mg^2+^, both conditions for which the chemical step appears to be rate-limiting (11, 12). The thio effect was compounded by an additional 4-fold for the *Rp* substrate vs. the *Sp* substrate (Fig. 3D), consistent with the coordination geometry seen in crystal structures (Fig. 3B). The interpretation of thio effects has long been a matter of debate in phosphoryl transfer catalysis, given the potential for complicating steric effects with phosphorothioate substrates (12, 13). Nevertheless, a plausible interpretation of the observed thio effects is that the chemical step sets the overall reaction rate for NP synthesis.

## Electrophilic substrate activation as a probable deficiency in NP catalysis with Ca^2+^

**Fig. 4.**
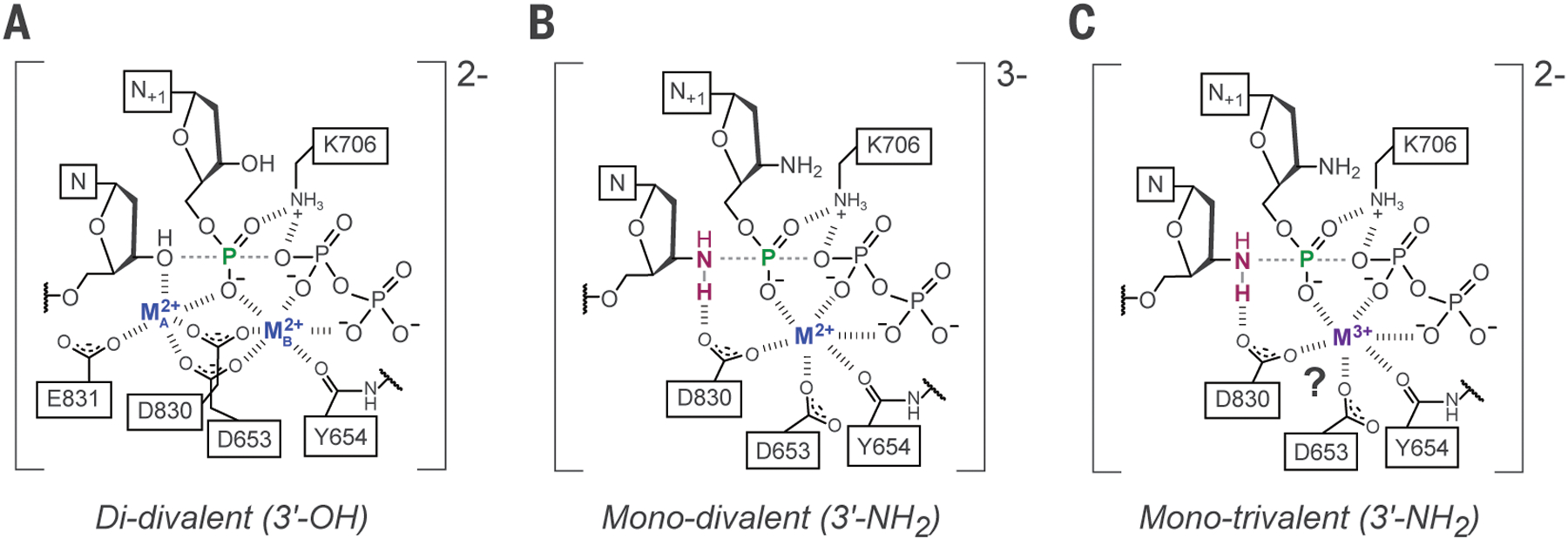
Comparison of reaction models for phosphodiester and phosphoramidate synthesis in BF. (**A**) Model for the canonical two-metal ion “di-divalent” mechanism for native phosphodiester activity. **(B**) Model for the mono-divalent mechanism for Ca^2+^-catalyzed NP activity. (**C**) Proposed mono-trivalent mechanism for REE-catalyzed NP activity. Note that neither inner sphere waters nor exact proton transfer pathways have been depicted. Two rate-limiting proton transfers have been detected for native activity (8, 11), but proton transfer is not rate-limiting for mono-divalent NP activity with Ca^2+^. At least one proton must nevertheless be transferred to generate the phosphoramidate product from the attacking neutral amine. In BF, these transfers likely involve deprotonated D830 as a general base and protonated K706 as a general acid.

If the chemical step is in fact rate-limiting but proton transfer is not, an augmented barrier to NP vs. phosphodiester synthesis could, at least in part, originate from an altered metal-ion configuration in the presence of a 3′-amino primer. In the native reaction center, it is generally understood that the A-site metal ion activates the 3′-OH nucleophile by inner sphere coordination, yielding a metal-alkoxide in the transition state, but numerous crystallographic models of reaction intermediates also show that the octahedral ion bound at the A site forms an additional inner sphere contact with the substrate pro-*Rp* α-phosphate non-bridging oxygen (14). It has been independently argued that the role of the conserved polymerase dinuclear metal center (Fig. 4A) is to stabilize the transition state electrostatically on the basis of linear free energy relationships (LFERs) obtained from Brønsted plots of pre-steady-state kinetics in polymerase *β*, harnessing a series of O_β,ɣ_ bridge substituent-modified substrate analogs with varying leaving group pK_a_ (15, 16). The catalytic effect of the A-site metal in native activity likely encompasses several effects, but any catalytic effect conferred by the presence of an A-site metal ion on substrate activation – as opposed to nucleophile activation – is absent for NP synthesis. The lack of electron density for an A-site metal ion in the ground state and at later time points is also consistent with the expectation of a neutral nucleophile in the NP reaction and the generally weak affinity of aliphatic amines for Ca^2+^. Since neither the apparent binding constant nor the active site conformation are significantly perturbed by the presence of a 3′-amino nucleophile (1), a significant contributor to the kinetic defect for NP vs. OP synthesis may therefore be the relative loss of transition state stabilization associated with the missing A-site metal cofactor for the reaction with an amino nucleophile.

If the native metal cofactors act, at least in part, on the native transition state by charge stabilization, it is noteworthy that the net effect of the absence of one divalent ion (with disengagement of its conserved ligand Glu-831) would be to decrease the formal net charge of the transition state complex by one (Fig. 4A,B). Outer sphere charge mutations may not be able to compensate for this inner sphere defect, particularly if the role of the missing metal in native activity is, in part, to contribute to stabilizing the developing charge polarization across the scissile bond. This view is consistent with the observation that eliminating the negative charge on the disengaged native ligand by the mutation E831Q fails to enhance NP synthesis kinetics (1).

## Trivalent metal ions confer rapid and specific NP synthesis activity

**Fig. 5.**
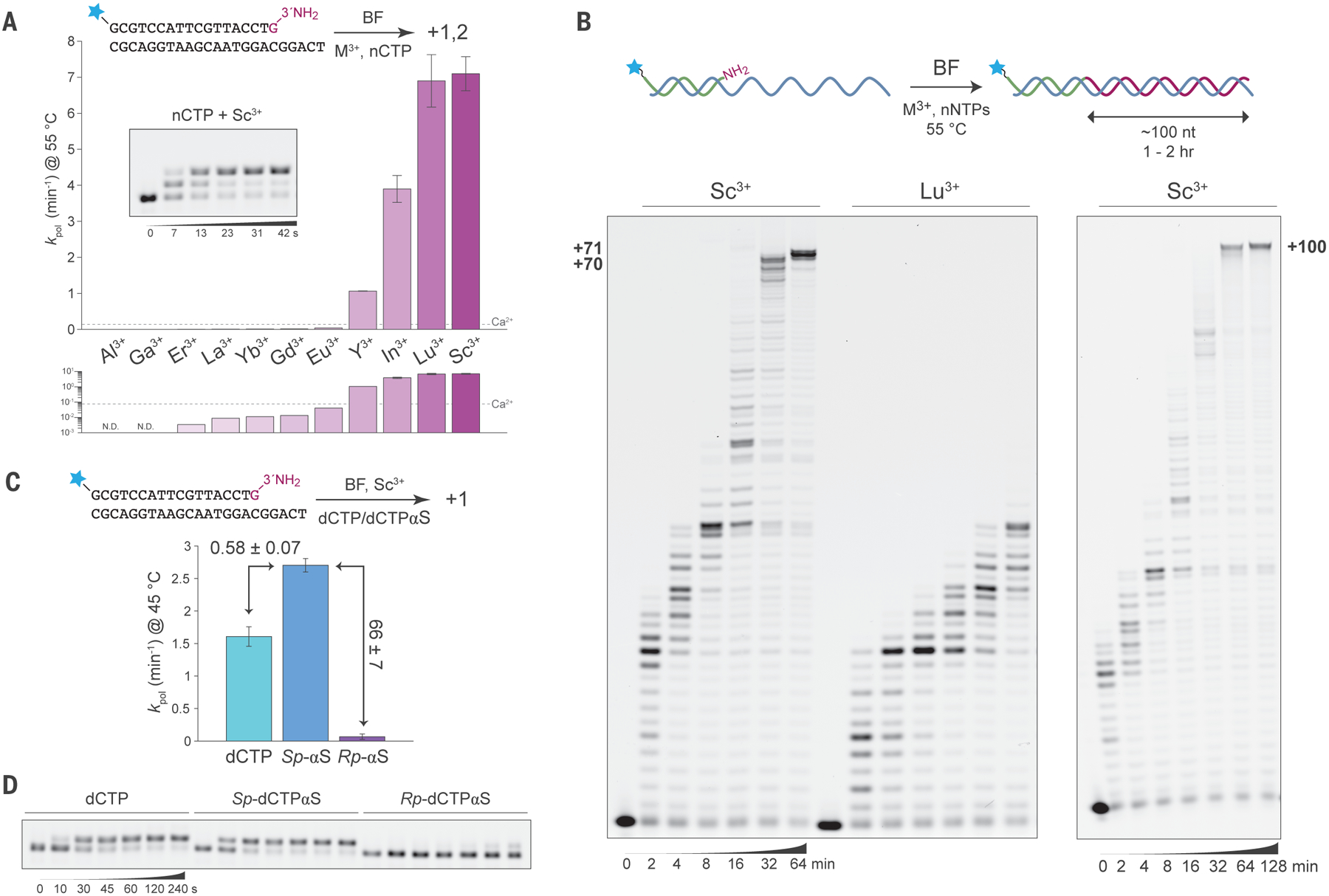
Trivalent rare earth metal ion cofactors confer rapid NP polymerase activity. (**A**) Pre-steady-state *k*_*pol*_ estimates for extension of a 3′-amino terminal DNA primer on a DNA template (inset cartoon) by BF F710Y/D598A at 55 °C in the presence of various 1:1 ammonium citrate-buffered trivalent metal cations at 5 mM in 40 mM Tris-HCl, pH 8.8, 2 mM βME, in reactions initiated by addition of 250 μM nCTP. Estimates are displayed in linear (top) and log scaled (bottom) axes in comparison with the level of Ca^2+^ activity (dotted line). Inset: representative 15% TBE-urea PAGE of quenched reaction samples from BF F710Y/D598A 3′-amino primer extension reactions in the presence of 5 mM Sc^3+^ showing +1 and +2 NP-DNA extension at the indicated times. N.D.: not detected. Error bars indicate s.e.m. for n = 4 (Sc^3+^ and Lu^3+^) or n = 3 (In^3+^ and Y^3+^). (**B**) Mixed sequence NP-DNA synthesis with excess BF F710Y/D598A on a 71-nt (left image) or 100-nt (right image) DNA templating region at 55 °C from a 5′-fluorescein-labeled 3′-amino-terminal DNA primer in the presence of 1:1 ammonium citrate-buffered 5 mM Sc^3+^ or Lu^3+^, as indicated, in 40 mM Tris-HCl, pH 8.8, 10 mM βME, and 25 μM spermine-HCl. Reactions were initiated by addition of 250 μM of each nNTP, sampled and quenched at the indicated times, and samples were separated on 10% (left) or 8% (right) TBE-urea PAGE gels. (**C**) Elemental thio effects (labeled arrows, mean ± s.d.) for Sc^3+^-activated NP synthesis with 500 μM *Sp*-αS, *Rp*-αS, or unmodified dCTP substrates at 45 °C estimated from *k*_*pol*_ (error bars indicate s.d., n = 3) using a fluorescently-labeled primer and template strand (cartoon at top), yielding exclusively +1 product due to high NP(S) specificity. (**D**) Representative 15% TBE-urea PAGE separation of quenched samples for the reactions in panel C.

On the basis of this activation argument and the observed thio effect, we hypothesized that substitution of a trivalent cation into the metal site B might compensate for the electrostatic component of the catalytic defect resulting from the absence of the A-site metal, following the hypothetical reaction structure depicted in Figure 4C. Upon screening a series of redox-stable trivalent metal ions, we indeed found that an exotic series of trivalent metal ions could act as polymerase cofactors for rapid catalysis of NP bond formation (Fig. 5). The diamagnetic group 3 trivalent rare earth element (REE) cations scandium (Sc^3+^), yttrium (Y^3+^), and lutetium (Lu^3+^), as well as the post-transition metal ion indium (In^3+^), all significantly improved single-nucleotide 3′-amino primer extension (Fig. 5A), as well as NP-DNA polymerization with all four nNTPs on mixed sequence templates (Figs. 5B, S3, and S4). Sc^3+^, in particular, accelerates pre-steady-state rate constants for nCTP addition by ~100-fold at 55 °C to 7.1 ± 0.5 min^−1^ (mean ± s.e.m) vs. 0.069 min^−1^ for Ca^2+^ (1), suggesting a stabilization effect of ca. −3 kcal/mol. Lu^3+^ yields similar levels of burst activity, *k*_*pol*_ = 6.9 ± 0.7 min^−1^ (mean ± s.e.m), but had far weaker multiple turnover activity in long primer extensions relative to Sc^3+^ (Figs. 5B and S3). NP-active trivalent ion cofactors have a wide range of pK_a_ values for the aquo ion, are prone to hydrolysis under the reaction conditions, and have a tendency to precipitate nucleotides and other phosphates. We found that reaction conditions were optimal when these ions were buffered at ~1:1 stoichiometry with citrate, such that all reaction components remain homogeneously soluble (Fig. S4). Although the reaction kinetics were quite sensitive to the metal:citrate stoichiometry, they were not appreciably sensitive to the concentration of 1:1 Sc^3+^:citrate in the low millimolar range (0.5 – 10 mM) in the presence of 1 mM total nNTPs (Fig. S4). However, it is known that the metal-ligand stoichiometry of trivalent REE citrate complexes in solution is diverse (17).

Trivalent metal ion cofactors confer exquisitely specific catalysis of NP vs. phosphodiester chemistry. We found that the pre-steady-state rate constant for extension of a native DNA 3′-OH terminus with nCTP in the presence of Sc^3+^ was 5.5 ± 0.6 × 10^−3^ min^−1^, which is at least three orders of magnitude slower than 3′-NH_2_ extension (Fig. S3B). NP-DNA synthesis activated by Sc^3+^ is also inhibited by added Mg^2+^ in the low millimolar range optimal for native activity (Fig. S5A). The mutation D830N is completely inactivating, whereas the adjacent E831Q mutant exhibits wild-type activity in long extensions (Fig. S5B), consistent with the active site configuration proposed in Fig. 4C. Another remarkable feature of trivalent NP catalysis is an apparent inverse elemental thio effect. We found that the Sc^3+^ thio effect was 0.58 ± 0.07 (mean ± s.d.) at 45 °C, indicating a significant preference for phosphorothioate substrates to yield thiophosphoramidate (NPS) linkages (Fig. 5C,D). Inverse thio effects are more readily explained in the context of inverted stereospecificity with thiophilic metal cofactor substitution, but in this case the catalyzed reaction remains highly specific (66-fold) for the *Sp* over *Rp* substrates at 45 °C. Synthesis of the potentially clinically-relevant NPS linkages under these conditions was confirmed by high resolution mass spectrometry (Fig. S6).

NP-DNA synthesis on long templates was aided by the addition of polyamines (Fig. S4). With spermine and Sc^3+^, full length products on mixed sequence DNA templates up to +71 nt or, with additional enzyme, up to +100 nt could be synthesized in 1 – 2 hr at 55 °C (Figs. 5B and S4). Polyamine rescue is consistent with linkage-dependent conformational effects on duplex helicity for NP-DNA:DNA hybrids that arise during long-range primer extension synthesis, or it might alternatively reflect competition for inhibitory metal coordination sites. Activation by spermine was optimal at low micromolar concentrations, consistent with its reported effects on Klenow activity previously attributed to helicity effects (18). Under these conditions, NP-DNA synthesis remains highly determined by template sequence, as omission of any single aminonucleotide from the nNTP mix dramatically stalls extension activity at or immediately before the first template position complementary to the missing substrate (Fig. S7A,B). Extension products also show characteristic features expected from the incorporation of unnatural NP linkages, such as acid lability at elevated temperature (Fig. S7A,B: HOAc lanes) and high resistance to 3′−5′ exonuclease I activity (Fig. S7C,D).

## Discussion

Unlike for native phosphodiester catalysis, only a single metal ion cofactor is detectable by crystallography during NP catalysis. Although transition states and other short-lived intermediates are of course not directly observable in our studies, the totality of the evidence supports a single metal ion mechanism with weakened substrate activation in 3′-amino primer extension by BF. From this inference, we identified a series of trivalent metal ion polymerase cofactors with dramatically enhanced reactivity and specificity for NP-DNA synthesis. Relative to the wild-type BF DNA polymerase, combining a single active-site point mutation with trivalent REE cofactor substitution accelerates NP synthesis by well over 1000-fold, yielding template-directed NP-DNA strands of useful lengths in benchtop enzyme reactions. In the presence of Sc^3+^ and spermine, overall NP polymerase reaction time to yield full length products (~0.9 – 1.3 min/nt at 55 °C) was similar to that reported for certain evolved xenonucleic acid (XNA) polymerases producing OP-linked strands incorporating synthetic sugars (0.75 – 1.5 min/nt at 50 – 65 °C (19)). REE-catalyzed NP polymerase reactions are therefore comparable to those of extensively mutated polymerases engineered for alternative phosphodiester synthesis activities by directed evolution (20). This finding suggests that the evolutionary distance to catalysis of distinct chemistry may be negligible when alternative cofactors are present, given a conservatively modified substrate. Recovering a conserved chemistry with highly divergent substrates, by comparison, may require a more extensive search of sequence space.

The considerable catalytic advantage conferred by trivalent metal ion cofactors implies that a broader family of polymerase activities may be accessible by directed evolution of substrate specificity, given the well-demonstrated ability of evolutionary methods to optimize native catalysis on XNA substrates (20, 21). NP-DNA falls within a class of phosphoramidate nucleic acids that have long been valued for their potential utility as nuclease-resistant antisense oligonucleotides (ASOs) (22). These polymers have also been extensively studied for their nonenzymatic self-assembly from chemically-activated phosphorimidazolide nucleotide analogs (23–26), on a path to developing synthetic protocells and model systems for key aspects of abiogenesis (27, 28). Despite lacking a 2′-OH group, short synthetic NP-DNA duplexes adopt a conformational geometry more similar to RNA than DNA (10), such that these polymers are viable templates for reverse transcriptase (RT) activity (29) but are poor substrates for several classes of nucleases (30, 31). Whether NP polymers are, like RNA, fully functional Darwinian polymers remains a matter of significant interest. The development of practical levels of NP polymerase activity is a crucial step on the path to demonstrating that this class of synthetic genetic materials is in fact functional and evolvable.

## Supplementary Material

Supplementary Material

## Data Availability

Crystallographic data and maps have been deposited into the PDB, under PDB codes 8SCG, 8SCI, 8SCJ, 8SCK, 8SCL, 8SCM, 8SCN, 8SCO, 8SCP, 8SCQ, 8SCR, 8SCS, 8SCT, and 8SCU. All other data are presented in the main text or supplementary materials.
