# Supplementary material for: Trivalent rare earth metal cofactors confer rapid NP-DNA polymerase activity
Source: Science. Author manuscript; Available in PMC 2024 Feb 23. (PMC10886449; doi:10.1126/science.adh5339)
Supplement: Supplementary Material [file NIHMS1967000-supplement-Supplementary_Material.pdf]

Science
AAAS

Supplementary Materials for

Trivalent rare earth element cofactors confer rapid NP polymerase activity

Victor S. Lelyveld, Ziyuan Fang, and Jack W. Szostak

Corresponding author: jwszostak@uchicago.edu

The PDF file includes:

Materials and Methods
Supplementary Text
Figs. S1 to S7
Tables S1 to S3

Materials and Methods

Materials

3′-Amino-2′,3′-dideoxynucleoside 5’-triphosphates were purchased from Trilink BioTechnologies. Stereopure 2′-deoxycytidine 5′-O-(1-thiotriphosphate), *Sp*- or *Rp*-dCTPαS, were obtained from Biolog Life Science Institute. PAGE purified 3′-amino terminal DNA oligonucleotide primers were prepared using 3′-amino-2′,3′-dideoxynucleoside 5′-phosphoramidites and reverse DNA phosphoramidites from Chemgenes, using oligonucleotide synthesis reagents from Glen Research. DNA template oligonucleotides were synthesized by IDT. Crystallization reagents were obtained from Hampton Research. High purity trivalent rare earth metal salts were obtained from Sigma Aldrich, Alfa Aesar, or Strem Chemicals. In particular, scandium(III) chloride hexahydrate, 99.9%, was obtained from Strem Chemicals. Invitrogen Dynabeads MyOne Streptavidin C1 magnetic beads were purchased from ThermoFisher. Tag-cleaved BF polymerase variants F710Y/D598A or D598A protein were prepared essentially as previously described (*1*), with minor modifications. Specifically, DH5α F′Iq cells (New England Biolabs) were used for protein expression for F710Y/D598A from a modified version of the plasmid in (*1*) incorporating the $lacI^q$ promoter mutation, pVSL10. Cell lysis was performed with NEBExpress Lysis Reagent (New England Biolabs) pH-adjusted to ~8.5 with KOH, otherwise as previously described (*1*). All other BF variants were prepared as in (*1*).

Time resolved X-ray crystallography and analysis

The conditions for BF polymerase catalyzed extension of a 3′-amino terminal DNA primer on a DNA template in intact crystals were assessed by bright field microscopy for their ability maintain crystal integrity over the full time course of bond formation, up to 24 hours for BF mutant F710Y/D598A and 48 hours for wild type (D598A) with reaction progress monitored by HPLC of redissolved crystals. The quaternary complex was assembled with protein, primer, template, Ca^{2+} ion, and 2′-deoxyguanosine 5′-triphosphate (dGTP) substrate and crystallized within 1 - 2 days under mild acidic conditions (pH 5 - 6) below the pK_a of the primer terminal 3′-amino group (~7.7 as the free aminonucleoside (*32*)), such that the extension reaction could be initiated simply by pH shift. Crystals were transferred from the mother liquor to a similar soaking solution at pH 8.8, then flash frozen in liquid nitrogen at a range of time points for subsequent data collection. The mother liquor for crystallization was 2 M ammonium sulfate, 0.1 M MES, pH 5.3 - 6.0, 20 mM $CaCl_2$, and 5% MPD mixed 1:1 with a protein-nucleic acid complexes prepared with 0.15 mM BF F710Y/D598A or D598A, 0.2 mM DNA template, 0.2 mM 3′-amino terminal DNA primer, and 10 mM dGTP, essentially as in (*1*). The composition of the soaking solution was 2 M ammonium sulfate, 0.1 M EPPS, pH 8.8, 20 mM $CaCl_2$, 5% MPD, and 10 mM dGTP.

Diffraction data were collected under a liquid nitrogen stream at 99 K at a wavelength of 1.0000 Å on Beamline 822 at the Advanced Light Source in the Lawrence Berkeley National Laboratory (USA) or at a wavelength of 1.033175 Å on Beamline 822 Beamline 23-ID-B at the Advanced Photon Source in the Argonne National Laboratory (USA). The crystals were exposed for 1 s per image with a 1 Å oscillation angle on Beamline 822 or 0.2 s per image with a 0.2 Å oscillation angle on Beamline 23-ID-B. The distances between detector and the crystal were set to 220–400 mm. The data were processed by HKL2000 (*34*). The structures were solved by molecular replacement by PHASER (*35*) using structure of 6UR9 as the searching model. All structures were refined by Phenix (*36*). After several cycles of refinement, some water molecules

were added in Coot (*37*). Data collection, phasing, and refinement statistics of the determined structures are listed in Tables S1 and S2. Although we observed two fully assembled complexes in the "closed" conformation in the asymmetric unit under these crystallization conditions, it was found during refinement that ground state B-factors were appreciably different between the two complexes, possibly due to distinctions in crystal packing and associated solvent channels in the crystal. Analyses performed here rely exclusively on data from the complex with lower B-factor (model chains A-C), although similar but much slower kinetic trends are observable in the second complex as well.

For kinetic analysis of crystallographic data, we adopted a method similar to that presented in Samara et al. (*33*), but with several key modifications. Briefly, we begin with a refined ground state model and then prepare difference maps ($F_O - F_C$) from each observed soaked crystal dataset vs. the ground state model, where structure factors have been scaled together using the CCP4 program SCALEIT essentially as described in (*6*). However, in this work we have not omitted any atoms from the reaction center in ground state model refinement. Since any model bias is time invariant, this approach better maintains consistent scaling for density changes, both positive and negative, across real space maps which would not otherwise be the case for dynamics occurring in unmodeled regions.

Pearson correlation maps were calculated by selecting the real space coordinates of the nascent bond (or any other point of interest) as a reference point and then calculating the pairwise correlation coefficient, r, of difference map density from n distinct $F_O - F_C$ difference maps across all points on the real space grid (x, y, z),

$$r(x,y,z) = \frac{\sum_i^n (\Delta\rho_{ref,i} - \overline{\Delta\rho_{ref}})(\Delta\rho_i(x,y,z) - \overline{\Delta\rho(x,y,z)})}{\sqrt{\sum_i^n (\Delta\rho_{ref,i} - \overline{\Delta\rho_{ref}})^2 \sum_i^n (\Delta\rho_i(x,y,z) - \overline{\Delta\rho(x,y,z)})^2}}$$

where $\overline{\Delta\rho_{ref}}$ is the mean difference map density at a reference point, $\overline{\Delta\rho}$ is the mean difference map density at lattice point (x, y, z), $\Delta\rho_{ref,i}$ is the density at the reference point in difference map i, and $\Delta\rho_i(x, y, z)$ is the density at grid point (x, y, z) in difference map i. The resulting correlation maps have r values on the interval [-1,1] and can be contoured for visualization at a desired correlation level to generate coefficient isosurfaces in which, for example, the correlation coefficient is greater than or less than a threshold value. Two-sided p-values were calculated by the *pearsonr* function of the Python *scipy.stats* package.

Linear regression "beta" maps were produced by least squares regression to estimate the slope β for the best fit line,

$$\boldsymbol{\Delta\rho_{ref}} = \beta\, \boldsymbol{\Delta\rho}(x,y,z) + \alpha\ ,$$

between observed density differences at a reference point, $\boldsymbol{\Delta\rho_{ref}} = [\Delta\rho_{ref,1}, \Delta\rho_{ref,2}, \ldots, \Delta\rho_{ref,i}]$, versus any "query" lattice point, $\boldsymbol{\Delta\rho}(x, y, z) = [\Delta\rho_1, \Delta\rho_2, \ldots, \Delta\rho_i]$, where the parameter α corrects for crystal-invariant (i.e. time-invariant) density differences across real space. Regression maps were then contoured for visualization at a desired β value to yield isosurfaces within which β is greater than or less than a desired threshold value. Regression statistics (e.g. coefficients of determination, R^2) or correlation coefficients, r, can also in principle be used for filtering β maps, but no such filtering was performed here. Regression and correlation analyses

of the spatiotemporal data are closely related, but they offer complementary information about the dynamics specifically relevant to bond formation without requiring more involved dimensionality reduction.

Similarly, observed first-order rate constant estimates, k_{obs}, were obtained by nonlinear regression of time-varying density differences $\boldsymbol{\Delta\rho}$ at any real space position (x, y, z) using the expression,

$$\boldsymbol{\Delta\rho}(x, y, z) = A\,(1 - e^{-k_{obs}\,\boldsymbol{t}})\ ,$$

for density differences calculated across maps, $\boldsymbol{\Delta\rho}(x, y, z) = [\Delta\rho_1, \Delta\rho_2, \dots, \Delta\rho_i]$, derived from crystals quenched at various times, $\boldsymbol{t}$, following initiation of the reaction by pH-shift. Here, A is scaling factor for the amplitude of the difference map density change observed at the point (x, y, z). Variance statistics were calculated from the regression covariance matrix. Baseline subtraction of density at $t = 0$ was performed prior to regression to correct for time-invariant model error.

<u>Pre-steady-state kinetic estimates</u>

Estimates of the pre-steady-state kinetic rate constants of BF-catalyzed primer extension were performed as previously described in (*1*) with minor modifications. Briefly, primer extension reaction mixtures containing 1 μM 5′-fluorescein labeled 3′-amino terminal primer, 1.25 μM DNA template, and 1 - 1.3 μM of BF F710Y were prepared in a buffer containing 40 mM Tris-HCl, pH 8.8, 2 mM β-mercaptoethanol (βME), and metal salts as indicated. The reaction was equilibrated at the indicated reaction temperature in a thermocycler for ~1 min, followed by initiation by rapid addition of nucleotide substrates. Reaction samples were taken manually at various times and rapidly quenched by 1:25 dilution into 98% formamide, 10 mM tetrasodium-EDTA. Reaction samples were denatured at 95 °C for 30 sec and cooled to room temperature prior to loading and separation on 15% TBE-urea polyacrylamide gels. Fluorescent bands were visualized on a laser scanning imager (Amersham Typhoon) and analyzed using the manufacturer's software.

For measurement of the elemental thio effect, reactions were carried out as above but at a reduced temperature of 45 °C due to challenges in manual sampling in the burst regime. For solvent deuterium kinetic isotope effect (SDKIE) measurements, reactions were carried out essentially as above with the following changes. First, stock reaction mixtures containing all indicated reaction components except enzyme and βME were lyophilized to dryness and reconstituted by addition of either water or 99.9% D_2O, followed by 10 mM βME from a 1 M stock solution prepared in either water or D_2O, and enzyme. For the D_2O reaction stock, the buffer was prepared at pH 8.62 prior to lyophilization, such that the final pD following resuspension was 8.8. Substrate stock solutions of 10 mM dCTP were prepared by resuspending extensively lyophilized substrate in either water or D_2O, and these stocks were in turn mixed at various ratios to furnish a series of substrate stocks with final D_2O contents of either 25, 50, 75, or 96%. Natural abundance or deuterated solvent reaction mixtures were similarly mixed at various ratios to yield a series of 9 μL reactions with final D_2O contents of 25, 50, 75, or 96% and equilibrated at 55 °C. Each reaction was initiated by addition of 1 μL of substrate at the same D_2O content, and the reactions were otherwise assayed as above. The pre-steady-state rate constant, k_{pol}, at saturating substrate concentration, as indicated, was estimated as the linear slope of the log-transformed fraction of remaining primer, $-ln(P/P_0)$ where P_0 is the initial primer amount, vs. reaction time at early times. Stocks of trivalent metal salts were prepared

from the metal trichloride hydrate in solution with ammonium citrate (1:1 stoichiometry on a metal:citrate basis) and pH-adjusted by addition of ammonium hydroxide. This stock was added to a final concentration of 5 mM on a metal-ion basis in enzyme reactions, except where indicated.

Long NP-DNA synthesis reactions

NP-DNA strands were generated by primer extension of a 5′-fluorescein labeled 3′-amino terminal primer on a synthetic DNA template. Reactions were carried out at 55 °C with 0.9 - 1 μM primer, 1.2 μM template, and 250 μM of each nNTP in a freshly-prepared NP synthesis buffer containing 40 mM Tris-HCl, pH 8.8, 10 mM βME, 25 μM spermine-HCl, and 5 mM of 1:1 ammonium citrate:$ScCl_3$, prepared as above. Longer extension reactions typically require excess BF protein, generally ~3 μM in the reaction. Samples of the reaction were taken at the indicated times and quenched by 1:50 dilution into 98% formamide, 10 mM tetrasodium-EDTA, denatured as above, and separated on TBE-urea gels. Addition of 1 μM unlabeled DNA, complementary to the template sequence, to the quenching buffer was used to promote labeled-strand separation when denaturating highly thermostable duplex products in the presence of diluted reaction salts.

Isolation of single-stranded extension products

Single-stranded primer extension products were prepared from primer extension reactions, performed as above, with the following modifications: a 3′-biotinylated DNA template oligonucleotide (+5 or +11 template sequence) was used, oligonucleotide concentrations were 2 μM primer and 2.5 μM template, and the reaction was incubated for 10 min at 55 °C in a total reaction volume of 20 μL. The extended primer strand was isolated using streptavidin-coated magnetic beads, followed by strand separation (see Fig. S7C). Briefly, 40 μL of streptavidin-coated magnetic beads from a commercial suspension at 10 mg/mL (Dynabeads MyOne Streptavidin C1) were washed briefly in 50 mM NaOH, 100 mM NaCl and re-separated using a magnetic rack, followed by three washes in the following washing buffer: 10 mM Tris pH 7.6, 100 mM NaCl, 5 mM tetrasodium EDTA, 0.1% (v/v) Tween 20 prepared in RNase-free water and filtered using a 0.2 micron sterile filter. The extension reaction was cooled to room temperature and quenched by addition of the washed magnetic bead suspension in wash buffer at a ~2:1 ratio by volume. Binding proceeded with slow rotation at room temperature for ~10 min - 1 hr, followed by at least three washes in the same buffer. The extended strand was eluted from the beads by pH-shift, as follows. After removing the supernatant from the final wash, 22.5 μL of 0.1 N NaOH was added, and the beads were gently resuspended and then immediately separated on a magnetic rack. The supernatant was transferred to a tube containing 5 μL of 1 M Tris-HCl pH 7.0. Elution from the beads was repeated with a second fraction of 22.5 μL of 0.1 N NaOH, and the two supernatants were then pooled for a total volume of 50 μL. The eluted oligonucleotide sample was then desalted using an Oligo Clean & Concentrator Kit (Zymo Research) using the manufacturer's protocol, with elution in 20 μL LCMS-grade water.

Mass spectrometry

Single-stranded reaction products were analyzed by high resolution liquid chromatography mass spectrometry (LC-MS), essentially as described in (*38*). Briefly, samples were injected onto an Agilent 1200 HPLC system with online diode array detector (DAD) coupled to an Agilent 6230 time of flight (TOF) spectrometer (Agilent Technologies) in negative ion mode equipped

with an electrospray ionization (Dual ESI) source with simultaneous coinjection of mass reference ions. Sample separation was performed using a 100 mm (length) × 1 mm (i.d.) XBridge C18 column with a particle size of 3.5 µm (Waters) at 50 °C in an ion pairing reverse phase chromatography system. The aqueous mobile phase (solvent A) was buffered with 200 mM hexafluoroisopropanol (HFIP) with 1.25 mM triethylamine (TEA) at pH 7.0 prepared in LCMS-grade water, and the organic mobile phase was LCMS-grade methanol (solvent B). Following mass axis calibration using the manufacturer's reference ion mix, desalted samples were loaded using an autosampler in 2.5 % solvent B isocratically for 4 min at a flow rate of 0.1 mL/min, with the first 2 min diverted from the MS instrument. Elution into the source was then carried out with the following gradient: 2.5 % to 15 % B over 16 min, 15 % to 40 % B over 21 min, followed by washing at 90 % B. Scans were collected from 239 to 3200 m/z at 1 spectrum/s. The mass analyzer settings were as follows: drying gas flow, 8 L/min; drying gas temperature, 325 °C; nebulizer pressure, 30 psig; capillary voltage, 3500 V; fragmentor, 200 V; and skimmer, 65 V. Data analysis was performed using the manufacturer's analysis software.

Nuclease resistance assay

Approximately 10 pmol of single-stranded oligonucleotide, prepared as above, was mixed with 1 µL of 10X Exonuclease I buffer (NEB) for a total volume of 9 µL using RNase-free water and equilibrated at 37 °C. Recombinant exonuclease I enzyme from *E. coli* (NEB) was diluted from the commercial stock in 1X Exonuclease I buffer to a concentration of ~1 U/µL, and the reaction was initiated by addition of 1 µL (1 U) of diluted enzyme. Samples were taken at the indicated times and quenched 1:10 in 98% formamide, 10 mM tetrasodium EDTA. Control oligonucleotides extended with DNA were prepared essentially as above, except using a 3′-OH DNA primer, 0.25 mM dNTPs, and 1X Thermopol buffer (NEB) containing 2 mM $MgSO_4$. Reaction samples were then separated and visualized by TBE-urea PAGE.

Supplementary Text

Comparative analysis of density dynamics in wild-type versus F710Y BF

We observed that the ground state substrate conformation is distinct between the mutant F710Y and wild-type BF. This raises the possibility that the role of the activating mutation F710Y could be, at least in part, to induce the observed C2′-endo substrate conformation. F710Y does modestly enhance the pre-steady-state rate constant for incorporation of deoxynucleotide substrates by ~2.5-fold in solution with a minor improvement in the kinetically-determined apparent dissociation constant, $K_{d,app}$ (*1*). However, the furanose moiety of 3′-amino nucleotides is generally understood to prefer the C3′-endo conformation due to a mixture of steric and anomeric effects. The 3′-amino sugar was indeed found as C3′-endo in the inactive reaction complex containing nGTP substrate and a dideoxy-terminal primer, as well as in the incorporated terminal 3′-amino product in a structure of the BF-bound translocated state following primer extension (*1*). Efforts to obtain crystals with 3′-amino groups on both the primer and substrate have so far been unsuccessful. If adoption of a C2′-endo conformation in the substrate were critical to the enhancing effect of F710Y, we would not expect such an enhancement for nNTPs. Yet the rate constant for incorporation of 3′-amino substrates was indeed enhanced by over 20-fold by the mutation, substantially more than for native deoxynucleotide substrates and with almost no effect on $K_{d,app}$. We therefore infer that the conformational shift of dGTP in reacting

BF F710Y crystals is unlikely to explain the enhanced kinetics for nNTP substrates afforded by the mutation.

Because this single activating mutation has such a large effect on the overall NP-DNA synthesis kinetics, we searched for alternative mechanistic explanations using a time-resolved dataset produced with the wild-type parent enzyme. As the reaction proceeds in wild-type crystals, a new region of density consistent with a solvent molecule accumulates within the Ca^{2+} ligand sphere (Ca^{2+}-H_2O distance ~3.2 Å), in a manner highly correlated with the formation of the nascent bond and concomitant with a subtle shift in the Ca^{2+} ion position (Fig. S2). The correlated emergence of this water in the wild-type active site (Fig. S2C, E) is not observed in the F710Y mutant, although density at the proximal β-phosphate substrate position becomes substantially disordered in both mutant and wild-type structures over time. In the mutant, a water at the equivalent site remains stably ordered throughout the reaction. One apparent consequence of the presence of this water is to alter the preferred Asp-653 ligand interaction from a predominantly bidentate to a monodentate configuration on the distal side of the ion (*39*). The coordination sphere ultimately found in the wild-type product state has therefore become more similar to the F710Y ground state, suggesting that the role of the activating mutation is to stabilize a favorable product-like metal-ligand geometry.

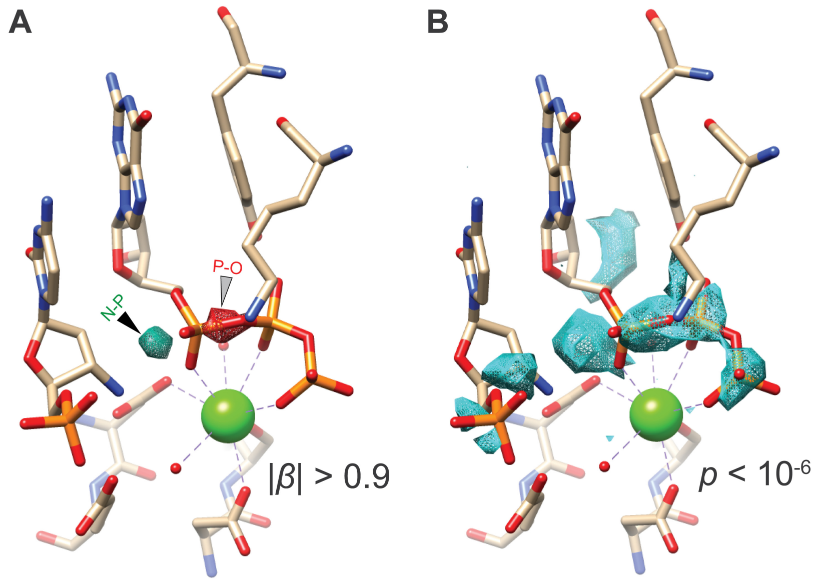

Fig. S1. Additional analysis maps of time-resolved NP extension in F710Y/D598A BF. (A) Linear regression map against the nascent bond (black wedge). Isosurfaces shown contoured at $|\beta| > 0.9$ and superimposed on the ground state model, as in Figure 2 of the main text. (B) P-value map for time resolved NP primer extension in F710Y/D598A BF. The P-value map was calculated as ($1 - p$) for the significance of pairwise correlation estimates in main text Figure 2, contoured at $p < 10^{-6}$, and presented as isosurfaces superimposed on the ground state model.

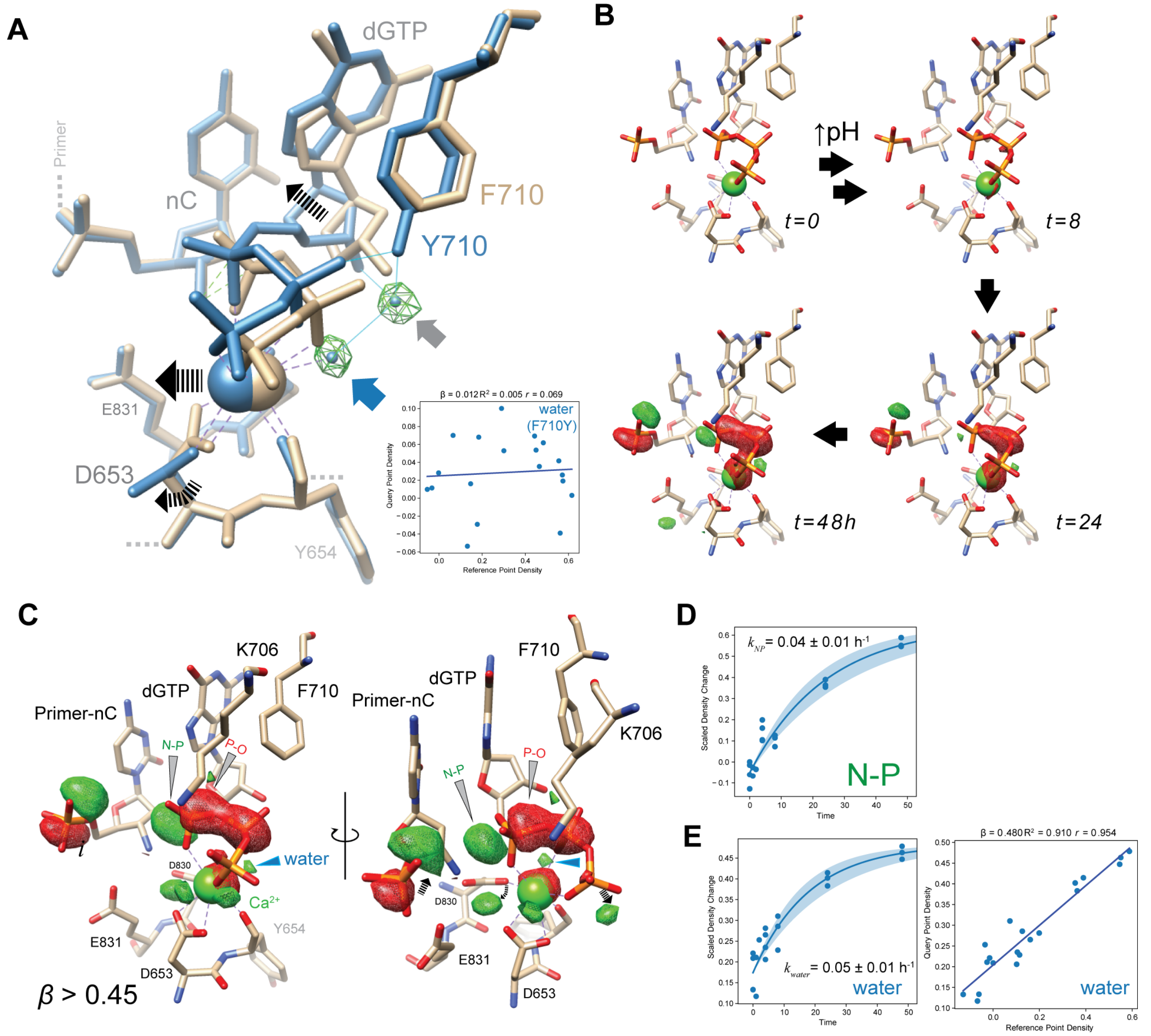

Fig. S2. Structural distinctions between BF F710Y mutant and WT in the ground state (GS) and during NP bond formation in crystallo. (A) Overlay of the active site in GS structures of F710Y and WT, both also incorporating the solvent-exposed crystal-contact mutation D598A, viewed from the perspective of the O-helix. Two ordered solvent molecules are apparent in the F710Y mutant, stabilized by the exocyclic hydroxyl of Y710 and the catalytic Ca^{2+} ion, with the resulting GS conformational distinctions indicated by black arrows. (B) Time resolved progress of NP bond formation in wild-type BF. Crystals of wild-type BF protein (containing the crystal contact mutation D598A) were prepared and soaked essentially as in Fig. 1 of the main text in a mother liquor containing $CaCl_2$ and dGTP, except soaking was carried out to 48 h. Difference maps are shown contoured at 3 σ, superimposed on the GS model, but time points up to 8 h show insignificant progress at this contouring and are not shown. (C) Voxelwise linear regression analysis of density dynamics during N3′→P5′ synthesis *in crystallo* in wild-type (D598A) BF. Pairwise linear regression map is calculated at all voxels vs. the site of the nascent NP bond (labeled gray wedge) with positive (green mesh) or negative (red mesh) regression slopes, β, displayed as isosurfaces superimposed on the GS structure model,

contoured at $|\beta| > 0.45$. Dashed arrows indicate conformational changes giving rise to the indicated high-beta lobes. (D) First-order kinetic regression of nascent NP bond in wild-type, as in main text Fig. 1, derived from difference map densities arising from 20 crystals. (E) Kinetic regression (left) and pairwise linear regression (right) for the nascent inner sphere water indicated in panel C (blue wedge).

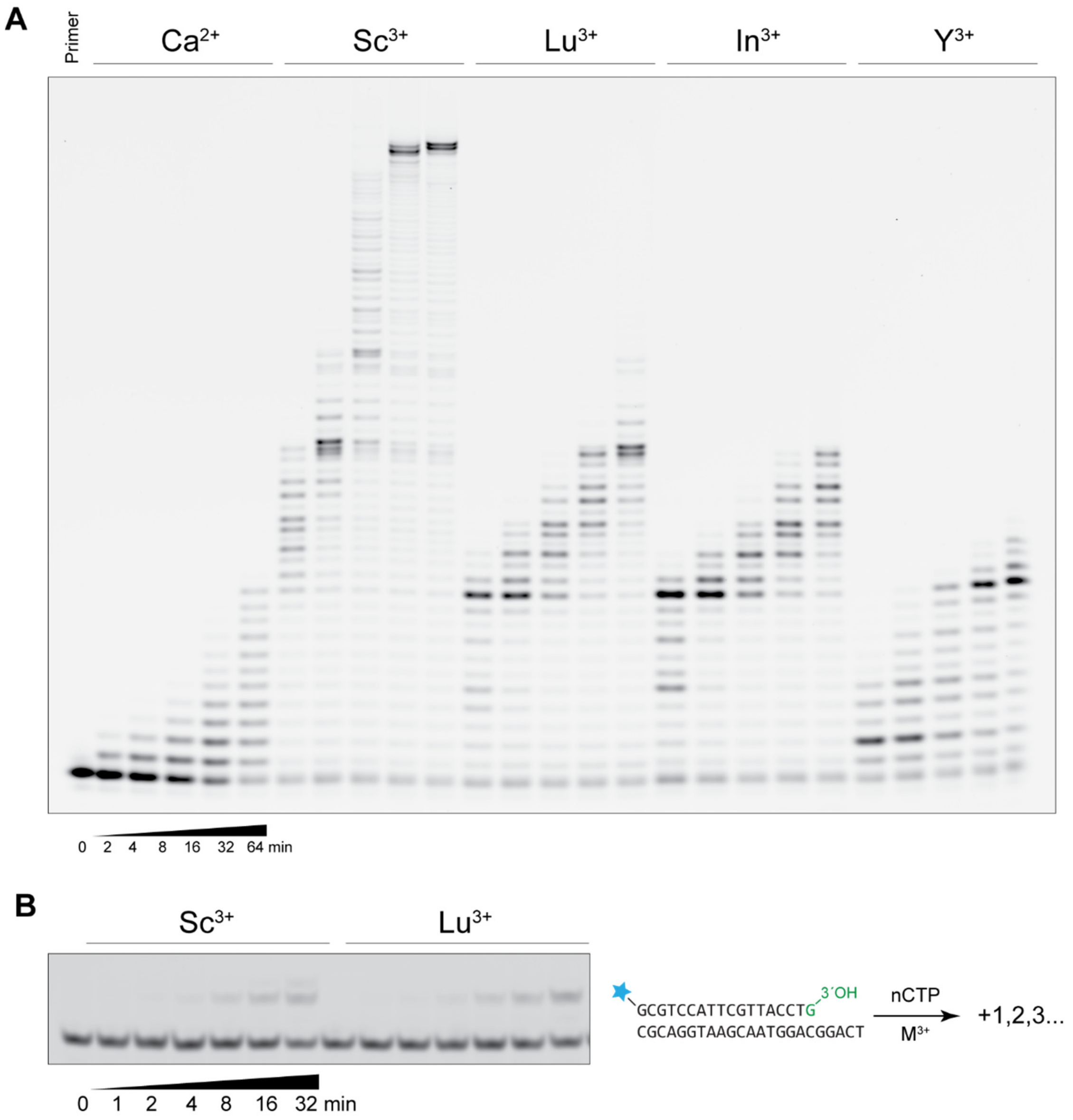

Fig. S3. Determinants of trivalent cofactor activity. (A) Multiple turnover extension in BF F710Y catalyzed by calcium chloride or various trivalent rare earth metal cofactors in the presence of stoichiometric citrate on a +71 nt DNA template. Conditions for extension were the same as in main text Fig. 5B, except with 10 mM $CaCl_2$ in lanes 2-6, 5 mM $ScCl_3$ and 5 mM ammonium citrate in lanes 7-11, 5 mM $LuCl_3$ and 5 mM ammonium citrate in lanes 12-16, 5 mM $InCl_3$ and 5 mM ammonium citrate in lanes 17-21, and 4 mM YCl_3 and 4 mM ammonium citrate in lanes 22-26. (B) Representative gel showing extension of a native DNA primer on a DNA template with nCTP and 5 mM Sc_{3+} or Lu_{3+} in the presence of stoichiometric citrate at 55 °C. Conditions are otherwise as in main text Fig. 3B. Estimated pre-steady-state rate constants were $5.5 \pm 0.6 \times 10^{-3}$ min^{-1} and $8 \pm 1 \times 10^{-3}$ for Lu^{3+}, respectively, from independent duplicates (mean ± s.d.).

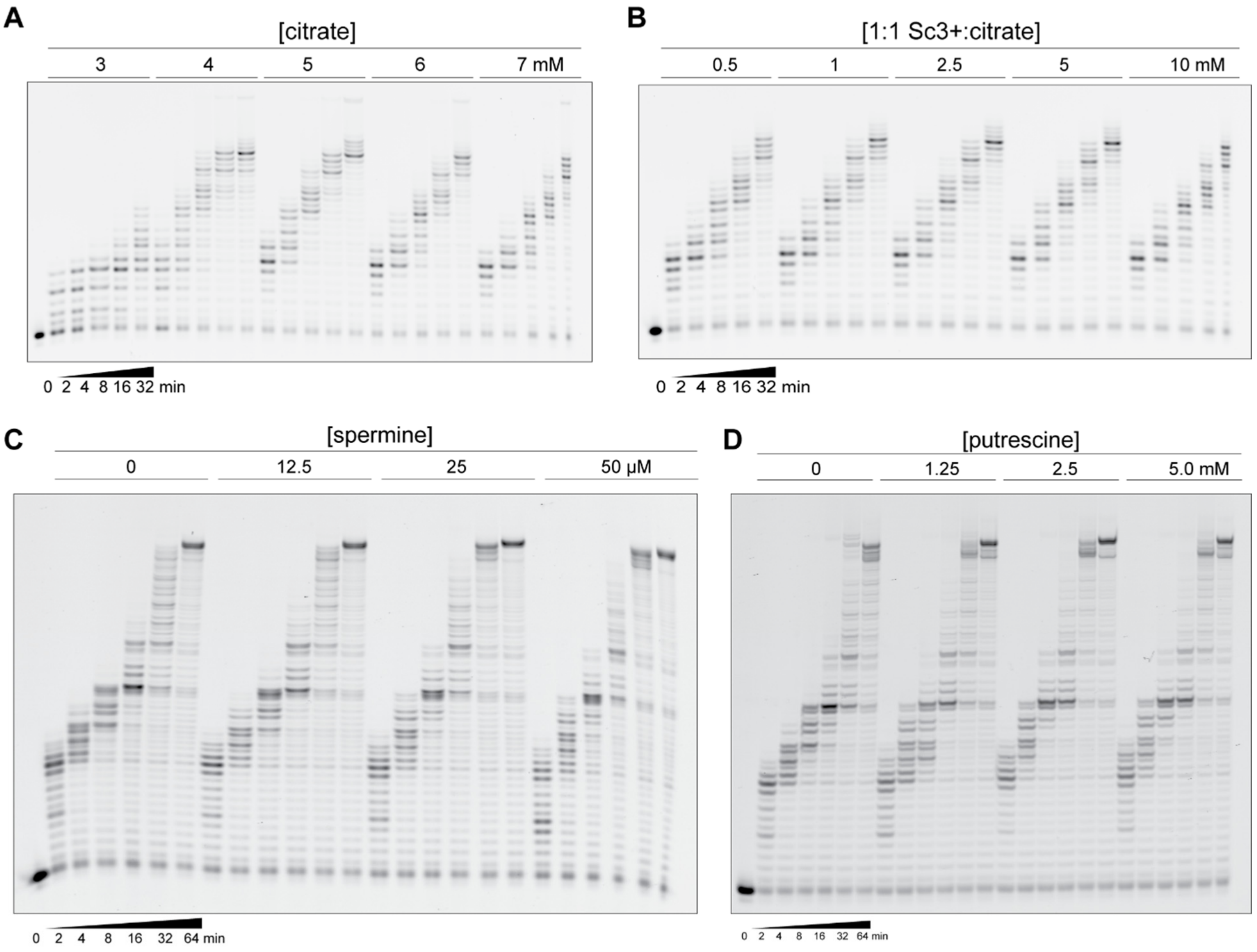

Fig. S4. Effects of metal citrate concentration and polyamines on multiple turnover NP polymerase activity. (A) Optimization of multiple turnover primer extension with BF F710Y protein and nNTP mix (250 µM each amino-nucleotide) and 5 mM $ScCl_3$ vs. various concentrations of ammonium citrate at 55 °C on a +28 nt DNA template. (B) Multiple turnover primer extension, as in panel A, with a range of metal concentrations and fixed stoichiometry of Sc^{3+}:citrate on a +28 nt DNA template. (C) Primer extension reactions as in main text Fig. 5B, except with spermine HCl at the indicated concentrations on a +71 nt DNA template. (D) Primer extension reactions as in panel C, but with the addition of putrescine HCl as the polyamine additive. The first lane of each gel is sampled from a fully assembled reaction master mix prior to addition of the indicated additive and nNTPs and therefore shows only unextended fluorescently-labeled primer. See Table S3 for oligonucleotide sequences.

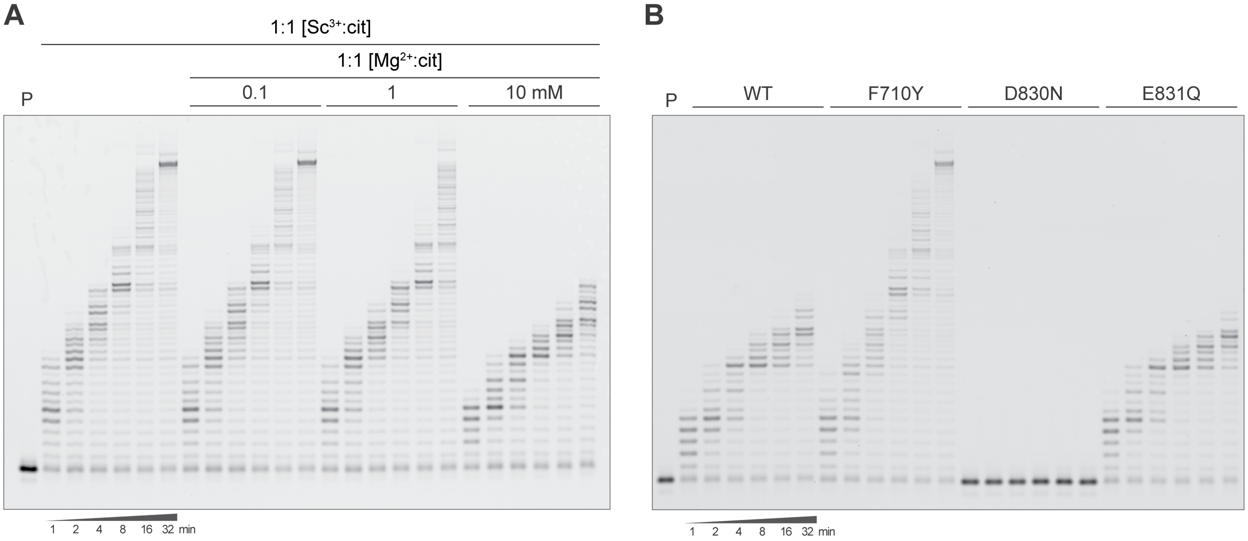

Fig. S5. Effects of Mg^{2+} and active site mutations on long NP-DNA primer extension activated by trivalent Sc^{3+}. (A) Primer extension reactions on the +71 template in the presence of 1:1 $ScCl_3$:ammonium citrate and the indicated concentrations 1:1 $MgCl_2$:ammonium citrate, at concentrations on the metal:citrate basis. Reaction samples were quenched at the indicated times, analyzed on a 15% TBE-urea gel, and otherwise carried out as in Fig. 5B of the main text. (B) Primer extension reactions as in Fig. 5B of the main text, except with the indicated BF polymerase active site mutants. The enzyme variant labeled as F710Y additionally carries the neutral crystal-contact mutation D598A.

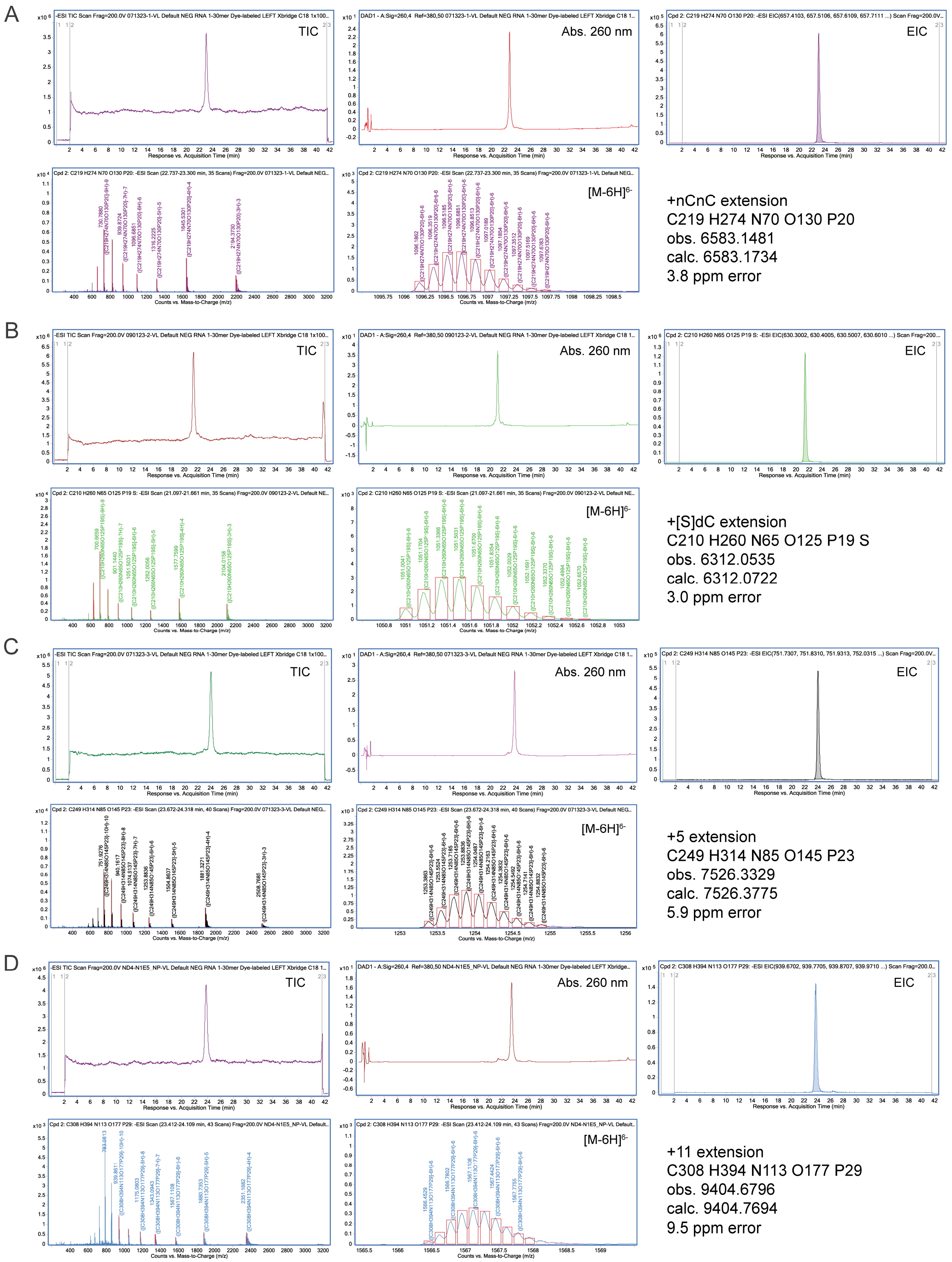

Fig. S6. High resolution LC-MS analysis of Sc^{3+}-catalyzed BF extension products. (A) Analysis of 3′-amino terminal DNA primer extended by two nC residues in a reaction containing

nCTP substrate on a 3′-biotinylated version of the template in main text Fig. 5A. (B) Analysis of 3′-amino terminal DNA primer extended by a thiophosphoramidate (NPS) linkage in a reaction with *Sp*-dCTPαS, as in main text Fig. 5C and D, on a 3′-biotinylated version of the template. (C) Analysis of 3′-amino terminal DNA primer extended by the sequence n[CCTGA] on a 3′-biotinylated +5 template in the presence of all four nNTP substrates at 250 μM. (D) Analysis of 3′-amino terminal DNA primer extended by the sequence n[CCTGATGCGTG] on a 3′-biotinylated +11 template in the presence of all four nNTP substrates at 250 μM. For all reactions, the extended single-stranded chimeric primer was isolated from the reaction as described in Materials and Methods. For all panels A-D, the insets show the total ion chromatogram (TIC), 260 nm UV absorbance chromatogram, extracted ion chromatogram (EIC) for the assigned chemical formula, the average spectrum with highlighted charge states for the salt adduct-free oligonucleotide (red bars with assigned charge state labels), and a magnified spectrum for an example charge state ($[M\text{-}6H]^{6-}$) with overlaid calculated isotope distributions for the indicated product chemical formula (red boxes).

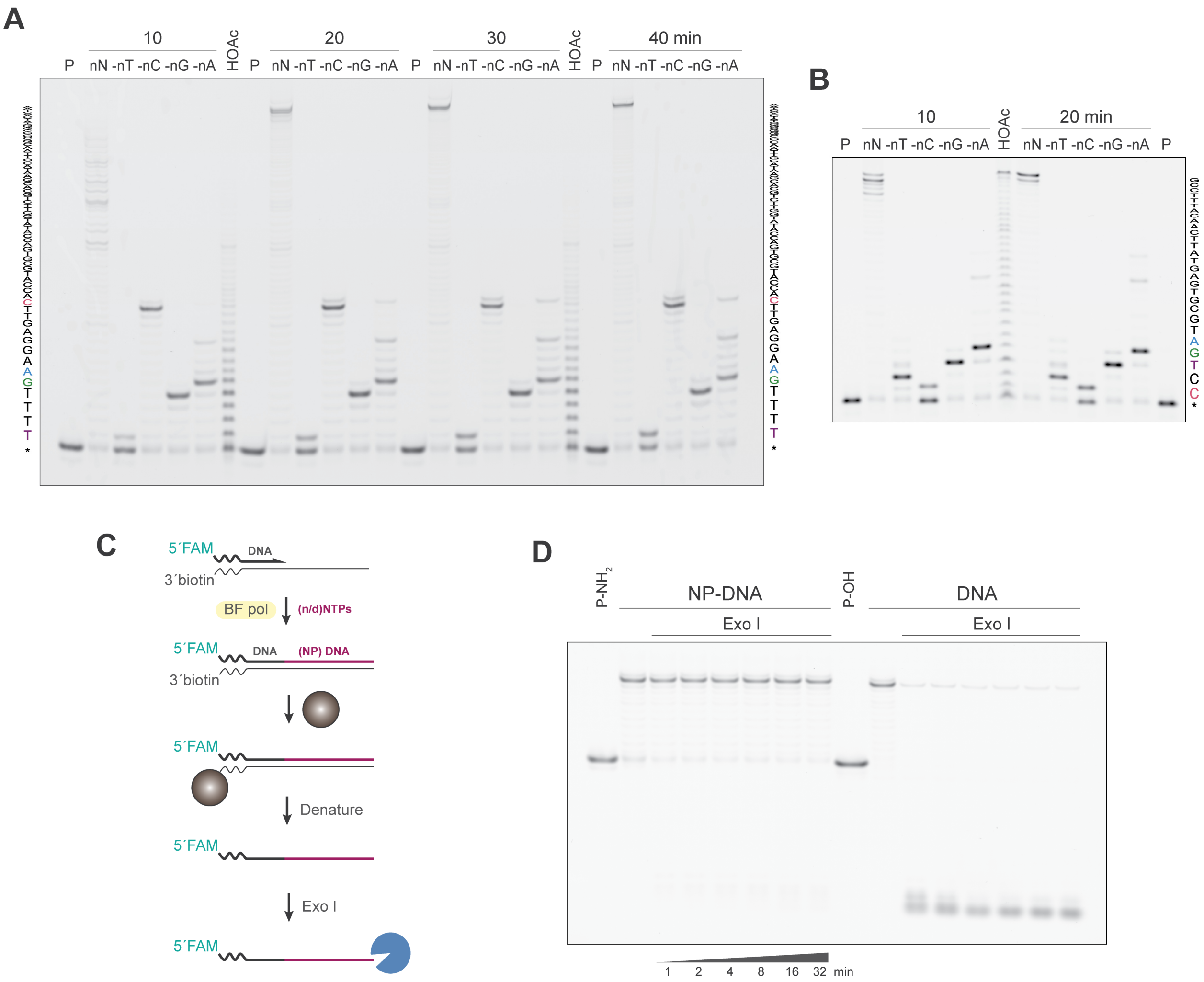

Fig. S7. NP-DNA primer extension activity is template-directed and yields nuclease-resistant products. (A) Primer extension reactions on the +71 nt template carried out as in Fig. 5B of the main text, except using single nucleotide drop-out substrate mixes where indicated. Reaction mixes equilibrated at 55 °C were initiated by addition of a substrate mix containing all four nNTP substrates (nN) or one prepared in the absence of the indicated aminonucleotide substrate. For each indicated reaction time (10 – 40 min), samples were quenched as in Fig. 5B. Partially hydrolyzed products are shown with hydrolytic fragments apparent only in the extended NP-DNA region of the chimeric product (HOAc lanes), prepared by incubating the full length extension product from the "nN" reaction 1:2.5 with 1% (v/v) HOAc in 98% formamide, 10 mM EDTA at 85 °C for 10 min (*1*). For all reactions, samples were quenched at the indicated times and then analyzed using a 15% TBE-urea gel (P: unextended primer). (B) Single-nucleotide drop-out reactions as in panel A, except prepared using the +28 template. (C) Preparation of a single-stranded chimeric DNA/NP-DNA product using a terminally-biotinylated template. Extension reactions were carried out using a biotinylated +11 template, otherwise as in Fig. S6, and the extended product duplex was isolated using a streptavidin-bound magnetic bead. After washing, the product strand was eluted using 0.1 N NaOH, followed by neutralization and desalting (see Materials and Methods). (D) Single-stranded products prepared as in panel C with either NP-DNA or DNA extensions were incubated with 1 U exonuclease I for the indicated

times at 37 °C. Samples were quenched and analyzed on a 15% TBE-urea gel (P-NH_2: unextended 3′ amino-terminal primer. P-OH: unextended DNA primer).

Table S1. Crystal X-ray data collection statistics

Item	F710Y 0h (GS)	F710Y 1h	F710Y 2h	F710Y 4h
PDB code	8SCG	8SCI	8SCJ	8SCK
Beamline	8.2.2	8.2.2	8.2.2	8.2.2
Wavelength (Å)	1.0000	1.0000	1.0000	1.0000
Space group	$P2_12_12_1$	$P2_12_12_1$	$P2_12_12_1$	$P2_12_12_1$
Unit cell parameters (Å, °)	93.68, 108.81, 149.55, 90, 90, 90	93.16, 108.54, 148.78, 90, 90, 90	93.03, 109.70, 149.84, 90, 90, 90	93.54, 108.97, 149.40, 90, 90, 90
Resolution range (Å)	50.00-2.00 (2.05-2.00)	50.00-2.68 (2.73-2.68)	50.00-2.67 (2.72-2.67)	50.00-2.30 (2.34-2.30)
Unique reflections	99476 (6410)	43114 (2134)	41492 (2033)	66713 (3344)
Completeness (%)	96.5 (88.4)	99.6 (100)	96.8 (97.3)	97.6 (100)
Redundancy	9.0 (7.8)	7.2 (7.3)	4.7 (4.8)	4.8 (4.9)
R_{merge} (%)	10.9 (44.9)	10.8 (46.3)	8.2 (45.3)	9.7 (43.2)
$\langle I/\sigma(I)\rangle$	16.7 (5.7)	16.4 (5.5)	14.8 (2.6)	12.7 (3.8)

Item	F710Y 6h	F710Y 8h	F710Y 24h (PS)
PDB code	8SCL	8SCM	8SCN
Beamline	8.2.2	8.2.2	8.2.2
Wavelength (Å)	1.0000	1.0000	1.0000
Space group	$P2_12_12_1$	$P2_12_12_1$	$P2_12_12_1$
Unit cell parameters (Å, °)	93.60, 109.74, 150.31, 90, 90, 90	93.97, 109.48, 149.82, 90, 90, 90	93.84, 108.79, 149.90, 90, 90, 90
Resolution range (Å)	50.00-2.44 (2.48-2.44)	50.00-2.30 (2.34-2.30)	50.00-2.30 (2.34-2.30)
Unique reflections	54903 (2768)	67705 (3421)	66403 (3372)
Completeness (%)	94.5 (97.5)	97.7 (100)	96.7 (100)
Redundancy	8.3 (8.4)	4.8 (4.8)	7.3 (7.2)
R_{merge} (%)	10.1 (49.2)	8.8 (49.8)	9.3 (48.3)
$\langle I/\sigma(I) \rangle$	16.7 (3.9)	14.4 (3.0)	21.7 (4.9)

Item	WT 0h (GS)	WT 1h	WT 2h	WT 4h
PDB code	8SCO	8SCP	8SCQ	8SCR
Beamline	23-ID-B	23-ID-B	23-ID-B	23-ID-B
Wavelength (Å)	1.033175	1.033175	1.033175	1.033175
Space group	$P2_12_12_1$	$P2_12_12_1$	$P2_12_12_1$	$P2_12_12_1$
Unit cell parameters (Å, °)	94.05, 109.04, 150.94, 90, 90, 90	93.86, 108.75, 149.99, 90, 90, 90	93.66, 108.55, 148.25, 90, 90, 90	93.77, 109.08, 151.18, 90, 90, 90
Resolution range (Å)	50.00-1.92 (1.95-1.92)	50.00-2.08 (2.12-2.08)	50.00-2.18 (2.22-2.18)	50.00-2.00 (2.03-2.00)
Unique reflections	111554 (4420)	90185 (4558)	78539 (3916)	101401 (5214)
Completeness (%)	93.6 (75.6)	97.0 (100)	98.8 (100)	96.3 (100)
Redundancy	6.3 (5.4)	6.0 (6.4)	6.1 (6.5)	6.4 (6.6)
R_{merge} (%)	5.5 (38.9)	7.9 (47.1)	9.5 (49.5)	4.8 (44.9)
$\langle I/\sigma(I)\rangle$	29.9 (4.1)	21.7 (4.2)	19.2 (4.1)	34.6 (4.2)

Item	WT 8h	WT 24h	WT 48h (PS)
PDB code	8SCS	8SCT	8SCU
Beamline	23-ID-B	23-ID-B	23-ID-B
Wavelength (Å)	1.033175	1.033175	1.033175
Space group	$P2_12_12_1$	$P2_12_12_1$	$P2_12_12_1$
Unit cell parameters (Å, °)	93.62, 108.68, 149.25, 90, 90, 90	94.32, 108.77, 149.57, 90, 90, 90	94.08, 109.16, 150.59, 90, 90, 90
Resolution range (Å)	50.00-2.10 (2.14-2.10)	50.00-2.34 (2.38-2.34)	50.00-2.38 (2.42-2.38)
Unique reflections	89558 (4432)	63660 (3215)	61520 (3062)
Completeness (%)	99.7 (100)	97.5 (100)	98.7 (99.8)
Redundancy	6.2 (5.9)	6.1 (6.5)	4.7 (4.7)
R_{merge} (%)	8.7 (50.5)	10.7 (48.5)	10.7 (49.7)
$\langle I/\sigma(I) \rangle$	19.4 (3.8)	13.9 (2.9)	11.8 (2.8)

Table S2. Crystal structure refinement statistics

Item	F710Y 0h (GS)	F710Y 1h	F710Y 2h	F710Y 4h
PDB code	8SCG	8SCI	8SCJ	8SCK
Resolution range (Å)	45.32 - 2.00	42.81 - 2.67	37.46 - 2.68	39.63 - 2.30
Number of reflections	96822	42855	40379	66267
R_{work} (%)	20.2	18.4	20.4	19.4
R_{free} (%)	24.5	25.6	26.0	24.8
Bond length R.M.S. (Å)	0.007	0.008	0.002	0.008
Bond angle R.M.S. (°)	0.91	0.96	0.51	0.91
Average B-factors (Å^2)	34.44	39.48	41.96	34.62

Item	F710Y 6h	F710Y 8h	F710Y 24h (PS)
PDB code	8SCL	8SCM	8SCN
Resolution range (Å)	40.98 - 2.44	44.10 - 2.30	44.11 - 2.30
Number of reflections	53540	65830	66193
R_{work} (%)	19.6	19.3	18.6
R_{free} (%)	26.8	25.00	24.3
Bond length R.M.S. (Å)	0.008	0.008	0.008
Bond angle R.M.S. (°)	0.96	0.93	0.90
Average B-factors (Å^2)	40.60	32.13	32.12

Item	WT 0h (GS)	WT 1h	WT 2h	WT 4h
PDB code	8SCO	8SCP	8SCQ	8SCR
Resolution range (Å)	41.52 - 1.92	39.86 - 2.08	46.83 - 2.18	44.39 - 2.00
Number of reflections	111220	90010	78029	100335
R_{work} (%)	18.9	18.9	18.8	19.4
R_{free} (%)	23.4	23.8	23.6	23.6
Bond length R.M.S. (Å)	0.007	0.007	0.008	0.008
Bond angle R.M.S. (°)	0.90	0.92	0.91	0.93
Average B-factors (Å^2)	26.31	30.37	34.53	31.65

Item	WT 8h	WT 24h	WT 48h (PS)
PDB code	8SCS	8SCT	8SCU
Resolution range (Å)	45.24 - 2.10	47.16 - 2.34	47.04 - 2.38
Number of reflections	89344	63462	60654
R_{work} (%)	19.0	18.7	18.7
R_{free} (%)	23.3	23.2	23.9
Bond length R.M.S. (Å)	0.007	0.007	0.008
Bond angle R.M.S. (°)	0.90	0.93	0.95
Average B-factors (Å^2)	30.05	35.31	42.06

Table S3. Oligonucleotide sequences

Oligonucleotide	Sequence (5′->3′)
Primer for crystallizations	GCGATCAGnC
Template for crystallizations	ACACGCTGATCGCA
Primer for solution phase reactions	Fluorescein-GCGTCCATTCGTTACCTnG
Template for solution phase kinetics	TCAGGCAGGTAACGAATGGACGC
Control primer with terminal 3′-OH	Fluorescein-GCGTCCATTCGTTACCTG
+5 template (3′ biotinylated)	TCAGGCAGGTAACGAATGGACGC-Biotin
+11 template (3′ biotinylated)	CACGCATCAGGCAGGTAACGAATGGACGC-Biotin
+28 template	CGGAAATGTTGAATACTCACGCATCAGGCAGGTAACGAATGGACGC
+71 template	TTAGGTGATACCCGCCGCGTTAACGTATTCGTGCAGAACCATATGGTCACGCATGGTGAACTCCTTCAAAACAGGTAACGAATGGACGC
+100 template	CATGTTGAATACTCACGCANNNNNNNNNNTTAGGTGATACCCGCCGCGTTAACGTATTCGTGCAGAACCATATGGTCACGCATGGTGAACTCCTTCAAAACAGGTAACGAATGGACGC

Note: Base codes marked with the prefix “n” are 3′-amino-2′,3′-dideoxynucleotides. All other codes correspond to standard DNA base codes.
